# Treating cat allergy with monoclonal IgG antibodies that bind allergen and prevent IgE engagement

**DOI:** 10.1038/s41467-018-03636-8

**Published:** 2018-04-12

**Authors:** J. M. Orengo, A. R. Radin, V. Kamat, A. Badithe, L. H. Ben, B. L. Bennett, S. Zhong, D. Birchard, A. Limnander, A. Rafique, J. Bautista, A. Kostic, D. Newell, X. Duan, M. C. Franklin, W. Olson, T. Huang, N. A. Gandhi, L. Lipsich, N. Stahl, N. J. Papadopoulos, A. J. Murphy, G. D. Yancopoulos

**Affiliations:** Regeneron Pharmaceuticals Inc., 777 Old Saw Mill River Road, Tarrytown, NY 10591 USA

## Abstract

Acute allergic symptoms are caused by allergen-induced crosslinking of allergen-specific immunoglobulin E (IgE) bound to Fc-epsilon receptors on effector cells. Desensitization with allergen-specific immunotherapy (SIT) has been used for over a century, but the dominant protective mechanism remains unclear. One consistent observation is increased allergen-specific IgG, thought to competitively block allergen binding to IgE. Here we show that the blocking potency of the IgG response to Cat-SIT is heterogeneous. Next, using two potent, pre-selected allergen-blocking monoclonal IgG antibodies against the immunodominant cat allergen Fel d 1, we demonstrate that increasing the IgG/IgE ratio reduces the allergic response in mice and in cat-allergic patients: a single dose of blocking IgG reduces clinical symptoms in response to nasal provocation (ANCOVA, *p* = 0.0003), with a magnitude observed at day 8 similar to that reported with years of conventional SIT. This study suggests that simply augmenting the blocking IgG/IgE ratio may reverse allergy.

## Abstract (web summary)

Allergen-specific immunotherapy is used to treat patients affected by acute immunoglobulin E (IgE) responses, but the function mechanism is unclear. Here the authors show that the administration of two cat allergen-specific IgGs reduces allergic responses in mouse models and helps ameliorate clinical symptoms in a phase 1b clinical trial.

## Introduction

Allergens are innocuous environmental or food substances that cause an inappropriate immune response resulting in allergic rhinitis, allergic asthma, food allergies, or atopic dermatitis in the allergic patient. Upon initial allergen exposure, antigen presenting cells capture, process, and present allergen peptides to cognate T-cells. In the presence of cytokines such as IL-4 or IL-13, T-cells acquire a type 2 phenotype, expand, and engage B cells to undergo class switch recombination resulting in polyclonal immunoglobulin E (IgE) production. IgE interacts with the high-affinity Fc-epsilon receptor (FcεR1) on the surface of mast cells and basophils to create “allergen receptors.” Allergen binding to IgE crosslinks the IgE:FcεR1 complex triggering degranulation and release of inflammatory mediators, termed the early phase response (EPR). The EPR occurs immediately after exposure and correlates with symptoms such as nasal congestion, rhinorrhea, sneezing, and itching^1^. Subsequent effector cell infiltration into the tissue comprises the late-phase response (LPR) along with continued production of IgE^1^.

Allergen-specific immunotherapy (SIT) is a treatment option for patients with allergic rhinitis triggered by aeroallergens (such as pollen, animal dander, or dust) when pharmacological therapies, such as antihistamines, are insufficient. SIT is also an investigational treatment for food allergies. SIT is an active immunization process whereby patients are administered increasing doses of the offending allergen, followed by a maintenance dose for up to several years. The goal of SIT is to induce immunological changes that result in symptom amelioration and sustained tolerance and desensitization. Although SIT can provide long-lasting protection from allergic disease, it carries a risk of adverse reactions due to administration of native allergen, has variable efficacy between patients, and can take 3–5 years to induce tolerance^2,3^.

Several hypotheses exist as to the underlying mechanism of successful SIT. One component is thought to be suppression of the effector T-cell response through the generation of allergen-specific regulatory T-cells^4^, allergen-specific T-cell anergy^5,6^, and a shift from Th2 cells toward Th1 cells^7^. Notably, a T-cell peptide vaccine seeking to recapitulate this mechanism failed in a Phase 3 study^8^. An alternative hypothesis is that SIT-induced allergen-specific IgG^9–12^ will compete with IgE for allergen binding, thereby decreasing allergen-induced effector cell activation. While titers of allergen-specific IgG generated through SIT have not consistently correlated with clinical improvement^13^, greater correlation is observed when comparing symptom improvement with the ability of blocking IgG to compete with IgE for allergen binding^9^ and functionally prevent the immediate hypersensitivity response^14,15^. These data suggest that augmenting the allergen-specific blocking IgG/IgE ratio is critically important to reducing allergic symptoms.

We sought to test the contribution of allergen-specific blocking IgG as the protective mechanism of SIT using pre-selected monoclonal anti-allergen-blocking antibodies to suppress allergic symptoms. To explore this fundamental question and test a therapeutic approach for allergy treatment, we generated two fully human IgG4 antibodies, REGN1908 and REGN1909, specific for Fel d 1, the major cat allergen^16^.

Here, we report that recombinant, allergen-specific blocking IgG antibodies perform comparably to allergen-specific IgG isolated from patients who completed successful SIT in preclinical studies. This effect translates to rapid and sustained reduction in clinical symptoms in patients with cat allergy undergoing nasal allergen provocation, thereby offering evidence that allergen-specific blocking IgG are an integral component of the protective mechanism of SIT, and may be a potential new and more rapid treatment approach for allergies.

## Results

### The potency of SIT-induced IgG is variable among patients

**Fig. 1:**
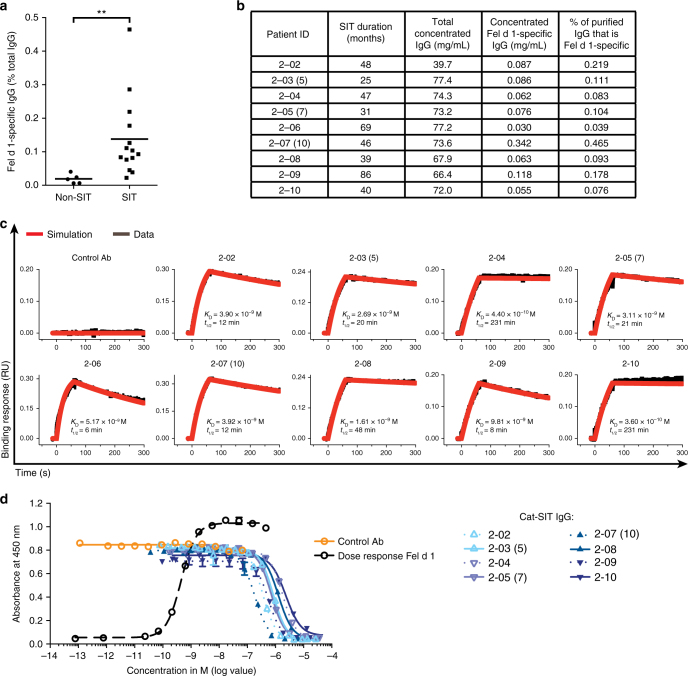
Specific immunotherapy (SIT) is associated with increased allergen-specific IgGs which have varying affinity between patients and block binding of Fel d 1 to IgE with variable potency. **a** Fel d 1-specific IgG titers were measured as a percentage of total IgG in cat-allergic Non-SIT (*n* = 5) and Cat-SIT (*n* = 14) patient sera by ELISA. Mean (line) and individual patient data representing the average of duplicate wells (dots) are shown. Statistical analysis was assessed by Mann–Whitney two-tailed test. **b** Individual patient data for samples used in (**c**) and (**d**). Patient IDs with parentheses denote three patients who donated blood at two visits. The number in parentheses represents donor ID from the first visit. Binding parameters presented here are based on sample obtained from their second visit. Samples from the first visit were used in the PCA Model, Fig. 4. **c** Purified and concentrated Cat-SIT-IgG samples from individual patients binding to rFel d 1 was assessed on a HTX biosensor platform. The rFel d 1.mmh was captured on anti-penta-His (HIS1K) Octet biosensors and dipped in samples containing 100 nM of Fel d 1-specific IgG. **d** Purified and concentrated Cat-SIT IgG from the individual patients shown in **b** and **c** were assessed for the ability to block binding of a constant concentration (0.7 nM) of rFel d 1.mmh from binding to plate-captured allergen-specific IgE from one cat-allergic patient in a blocking ELISA. One of two experimental replicates using different IgE donors is shown. The mean and SD from duplicate wells are plotted. Donor 2–06 was not included in the ELISA assay due to volume requirements of the assay. **p< 0.01

To characterize the IgGs induced by SIT, we obtained sera from patients with cat allergy who underwent successful immunotherapy (Cat-SIT) (range: 13–86 months; median: 33 months) and compared the IgG to cat-allergic control patients (Non-SIT) IgG in a series of in vitro assays. Using ELISA we confirmed that Cat-SIT patients had a higher percentage of Fel d 1-specific IgG compared to Non-SIT patients with cat allergy (Fig. 1a). Polyclonal Fel d 1-specific IgG (Cat-SIT-IgG) from nine patient samples (Fig. 1b) bound recombinant Fel d 1 produced with a myc–myc–hexahistidine tag (rFeld1.mmh), with apparent K_D_ values ranging from 0.36 to 9.8 nM (Fig. 1c, Supplementary Table 1). We also examined the ability of Cat-SIT-IgG to block Fel d 1 binding to cat-allergic patient IgE in a blocking ELISA. Cat-SIT-IgG blocks rFel d 1.mmh binding to IgE to baseline; however, the blocking potency is low (IC_50_ values ranging from 0.18 to 2.3 μM, Fig. 1d). Duration of Cat-SIT (Fig. 1b) did not correlate with Fel d 1-specific titers, affinity, or potency. Collectively, these data suggest that Cat-SIT induces an increase in circulating Fel d 1-specific IgG, which can bind to Fel d 1 with varying kinetics, but, as a whole, Cat-SIT-IgG inefficiently blocks Fel d 1 binding to polyclonal patient IgE.

### Together REGN1908 and REGN1909 block Fel d 1 binding to IgE

**Fig. 2:**
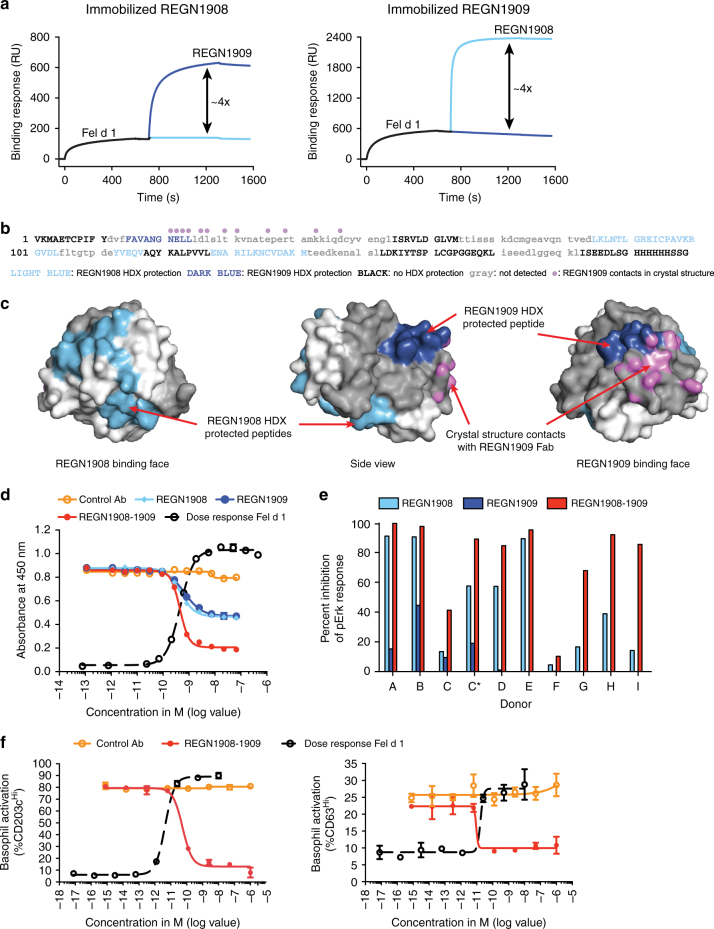
REGN1908 and REGN1909 simultaneously bind to distinct Fel d 1 epitopes and prevent Fel d 1 binding to Fel d 1-specific IgE. **a** Non-competitive binding of REGN1908 and REGN1909 was assessed by first injecting nFel d 1 over a CM5 sensor surface immobilized with REGN1908 (left) or REGN1909 (right), followed by the injection of REGN1908 (light blue) or REGN1909 (dark blue). **b** Using hydrogen/deuterium exchange (HDX), REGN1909 protected a single peptide region in rFel d 1 (dark blue) corresponding to amino acids 32–41 of Chain 2. REGN1908 (light blue) protected two peptide regions corresponding to Chain 1 amino acids 43–47 and 57–71. Light gray lowercase sequence denotes regions not detected by HDX. Violet dots denote residues making contact with REGN1909 Fab as shown in **c**. **c** The crystal structure of rFel d 1 bound to the REGN1909 Fab is shown with chain 1 surface colored in white and chain 2 colored in gray. The HDX-protected residues are colored light blue and dark blue as in (b). Atoms making contact (distance <3.5 Å) with the REGN1909 Fab are colored violet. **d** Representative ELISA using IgE from 1 of 4 donors testing antibody blocking of 0.7 nM rFel d 1.mmh from binding to Fel d 1-specific IgE is shown with mean and SD plotted. **e** REGN1908, REGN1909, or REGN1908–1909 blocking of 200 pM nFel d 1-induced basophil activation from cat-allergic donors, measured by flow cytometry and shown as percent maximum inhibition of pErk response relative to isotype control antibody. C and C* represent independent blood donations from the same donor 2 months apart. Individual donor and antibody dose–response data are shown in Supplementary Fig. 2. **f** REGN1908–1909 blocking of 20 pM nFel d 1-induced basophil upregulation of CD203c^hi^ (left) or CD63^hi^ (right) was measured by flow cytometry. Average value of duplicate wells and SD are shown. One representative donor out of 8 tested and 1 of 4 are shown for CD203c and CD63 activation, respectively. For combination studies REGN1908–1909 are combined in a 1:1 molar ratio and total antibody is plotted. Flow cytometry gating strategies are shown in Supplementary Fig. 5

To determine whether monoclonal antibodies could block allergen binding to IgE as well or better than those naturally produced during SIT, we generated Fel d 1-specific, fully human monoclonal antibodies using Regeneron’s VelocImmune human antibody mouse platform^17,18^. Given the polyclonality of allergen-specific IgE response, we hypothesized that multiple epitopes on the allergen would need to be blocked to prevent IgE-mediated effector cell activation. Therefore, we screened more than 300 antibodies, and selected two antibodies, REGN1908 and REGN1909, with optimal binding and blocking profiles for development. REGN1908 and REGN1909 were produced with an IgG4 constant domain containing a serine to proline amino acid substitution (S228P, EU numbering) in the hinge region that reconstructs the human IgG1 hinge sequence (CPPC) to promote stabilization of disulfide bonds between the two heavy chains^19^, designated IgG4^P^. Surface plasmon resonance (SPR)-Biacore binding studies demonstrated that REGN1908 and REGN1909 each bound with subnanomolar affinity to rFel d 1.mmh and natural Fel d 1 (nFel d 1), and sequential binding assays confirmed independent, non-competitive binding to nFel d 1 (Fig. 2a, Supplementary Fig. 1). Hydrogen/deuterium exchange (HDX) epitope mapping experiments indicated that REGN1909 binds Chain 2 of Fel d 1, while REGN1908 binds a region in Chain 1 on the opposite side of the molecule (Fig. 2b). The Fel d 1 peptide regions protected from amide exchange by REGN1908 and REGN1909 revealed a discontinuous epitope for REGN1908, predominantly in Chain 1, and a single linear epitope for REGN1909 in Chain 2 (Fig. 2b). To further refine the specificity of REGN1909 binding, an X-ray crystal structure of the Fab fragment of REGN1909 bound to Fel d 1 was resolved. This structure revealed that REGN1909 contacts multiple residues in the HDX-protected peptide (Fig. 2b, c), along with several additional contacts to Fel d 1 residues in a Chain 2 peptide not detected by HDX. Crystals were unable to be obtained for the REGN1908 Fab bound to Fel d 1. Together these data support a model in which REGN1908 and REGN1909 bind simultaneously and non-competitively to conformational epitopes at opposing regions on the surface of the Fel d 1 heterodimer.

REGN1908 and REGN1909 were evaluated individually and in combination for the ability to block Fel d 1 binding to cat allergen-specific polyclonal human IgE. In a blocking ELISA, each antibody partially blocked nFel d 1 binding to IgE with a maximum blockade of 51%, whereas the combination of REGN1908 and REGN1909 (REGN1908–1909) increased maximum blockade to 83% with an IC_50_ value of 0.45 nM. Next, we tested the ability of REGN1908–1909 to block Fel d 1-induced activation of human basophils from cat-allergic donors using two different assays (Supplementary Fig. 5). To assess FcεR engagement and activation, basophils were tested in a functional phosphoflow assay that measures phosphorylation of the kinase ERK, a proximal readout of basophil activation and degranulation^20^. Basophils from all 10 cat-allergic donors responded to Fel d 1 stimulation with varying intensities (Supplementary Fig. 2). REGN1908–1909 inhibited at least 80% of basophil activation in 7/10 donors, while a single antibody (REGN1908) achieved the same magnitude of inhibition in only 3/7 donors (Fig. 2e, Supplementary Fig. 2). In the basophil activation test (BAT), which measures allergen-induced upregulation of the basophil activation markers, CD203c and CD63, by flow cytometry^21^, REGN1908–1909 potently blocked Fel d 1-induced basophil activation (IC_50_ values of 55pM and 10 pM, respectively) (Fig. 2f).

These data establish that REGN1908 and REGN1909 bind with high affinity, simultaneously, and in a non-competing fashion to Fel d 1, and are more effective in combination when blocking Fel d 1 binding to polyclonal IgE from patients with cat allergy.

### REGN1908–1909 inhibits mast cell degranulation in vivo

**Fig. 3:**
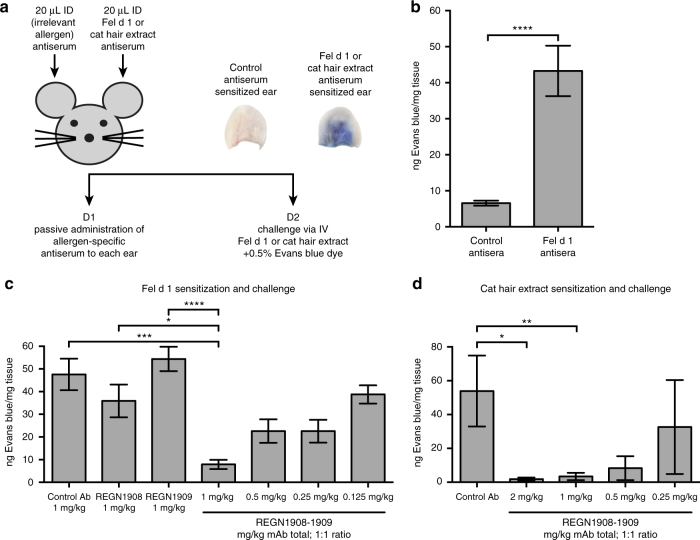
A combination of REGN1908–1909 inhibits Fel d 1-induced mast cell degranulation in vivo in the passive cutaneous anaphylaxis (PCA) mouse model. **a** Schematic of the PCA mouse model. **b** Each mouse (*n* = 5 in two independent experiments) were sensitized using Fel d 1 antisera and control peanut antisera in the left and right ears, respectively, followed by challenge with 1 μg nFel d 1 one day later. Data from the pooled experiments are expressed as ng of Evans blue extracted per mg of ear tissue from ears sensitized with control antisera or ears sensitized with peanut antisera. Statistical analysis was performed using a two-tailed paired *t*-test. **c** Balb/c mice were administered subcutaneous (SC) doses of REGN1908 (*n* = 15), REGN1909 (*n* = 15), a combination of REGN1908–1909 (1:1 ratio) at 0.125 mg/kg (*n* = 15), 0.25 mg/kg (*n* = 15), 0.5 mg/kg (*n* = 15), or 1 mg/kg (*n* = 14), or an IgG4^P^ control antibody (*n* = 14) 3 days prior to initiating the PCA model using Fel d 1-specific antisera (normalized to 25ng IgE per ear) and 1 µg Fel d 1 challenge. The number per group represents pooled data from three independent experiments. **d** Balb/c mice were administered SC injections of a combination of REGN1908–1909 (1:1 ratio) at 0.25 mg/kg (*n* = 5), 0.5 mg/kg (*n* = 5), or 1 mg/kg (*n* = 5), or 2 mg/kg (*n* = 5), or an IgG4^P^ control antibody (*n* = 5) 3 days prior to initiating the PCA model using cat extract antisera normalized to 25ng IgE per ear and 250 BAU cat hair extract challenge. Results of one experiment are shown. **c**, **d** Data are expressed as ng of Evans blue extracted per mg of ear tissue based on the value obtained from each control peanut ear subtracted from the corresponding value from the challenge ear. Statistical significance was determined using the Kruskal–Wallis test followed by the Dunn’s post hoc test (**c**–**d**). Data are presented as mean ± SE. **** *p* < 0.0001, ***p < 0.001, **p < 0.01, *p < 0.05

The potency of REGN1908 and REGN1909 was evaluated in vivo using the passive cutaneous anaphylaxis (PCA) mouse model with either nFel d 1 or cat hair extract, which contains a heterogeneous mixture of cat proteins including Fel d 1 (Supplementary Fig. 3). The PCA model assesses type 1 hypersensitivity and measures local mast cell activation-induced vascular permeability in ear tissue^22^ (Fig. 3a). After passive sensitization with nFel d 1 mouse antisera or control antisera, Fel d 1 challenge induced significant mast cell degranulation only in the nFel d 1 sensitized ear (*p* < 0.001, by Kruskal–Wallis followed by Dunn’s post hoc test, Fig. 3b), validating the model and confirming specificity of the allergic response.

The combination of REGN1908–1909 significantly blocked nFel d 1-induced mast cell degranulation at a dose of 1 mg/kg (total antibody; 1:1 ratio,  *p* < 0.001, by Kruskal–Wallis followed by Dunn’s post hoc test, Fig. 3c). Notably, the combination demonstrated a synergistic effect—only 0.5 mg/kg of each antibody was required to block degranulation when combined, whereas 1 mg/kg of each antibody alone had a minimal effect (Fig. 3c). In a similar model using cat hair extract, REGN1908–1909 also blocked cutaneous anaphylaxis (Fig. 3d), establishing that Fel d 1 predominately drives cat hair extract-induced mast cell degranulation in this model. These data reveal that REGN1908 and REGN1909 must be administered together to achieve efficacy in the PCA model, demonstrating that multiple Fel d 1 epitopes must be bound to prevent Fel d 1-induced crosslinking of polyclonal IgE and subsequent effector cell activation.

### REGN1908–1909 is more potent than Cat-SIT-IgG in vivo

**Fig. 4:**
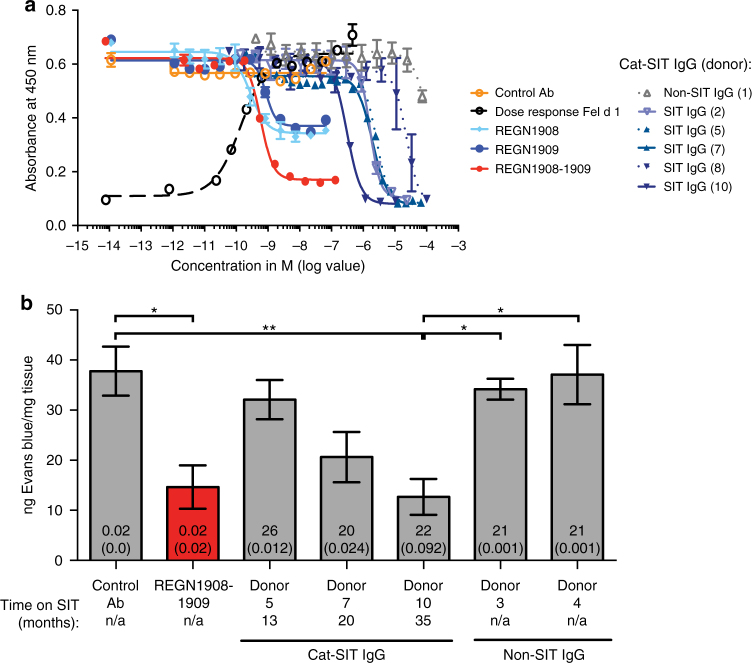
REGN1908–1909 blocks Fel d 1 binding to polyclonal Fel d 1-specific IgE more efficiently than natural IgGs from specific immunotherapy (SIT) patients. **a** In a blocking ELISA, the ability of REGN1908, REGN1909, REGN1908–1909, or Cat-SIT-IgG to block 0.7 nM rFel d 1.mmh from binding to plate-captured Fel d 1-specific IgE from one donor was assessed. A representative experiment using IgE from 1 donor of 3 donors tested is shown. The mean and SD from duplicate wells are plotted. **b** Balb/c mice were administered subcutaneous (SC) injections of a combination of REGN1908–1909 (1:1 ratio, *n* = 10), an IgG4^P^ control antibody (*n* = 10), or concentrated Cat-SIT-IgG from donor 5 (*n* = 9), donor 7 (*n* = 10), donor 10 (*n* = 10) or Non-SIT-IgG from donor 3 (*n* = 10) or donor 4 (*n* = 10) prior to the passive cutaneous anaphylaxis model using nFel d 1. Donors 5, 7, and 10 correspond to Donors 2-03(5), 2-05(7), and 2-07(10), respectively in Fig. 1 and Supplementary Table 1. The number per group represents pooled data from two independent experiments. Data are presented as mean ± SE ng of Evans blue extracted per mg of ear tissue is based on the value obtained from each control peanut ear subtracted from the corresponding value from the challenge ear. The total IgG concentration injected (mg), and Fel d 1-specific concentration injected (mg) is represented by the numbers on the bar graphs, with the latter in parentheses. Statistical significance was determined using the Kruskal–Wallis test followed by Dunn’s post hoc test. **p < 0.01 or *p < 0.05

To compare the ability of REGN1908–1909 or Cat-SIT-IgG to inhibit Fel d 1 from binding to polyclonal IgE, we performed both a blocking ELISA and the PCA mouse model. Both REGN1908–1909 and Cat-SIT-IgG blocked Fel d 1 binding to donor polyclonal cat-specific IgE (Fig. 4a). Cat-SIT-IgG blocked binding to baseline, but a high IgG concentration was required (IC_50_ range: 0.309–23.15 µM). In contrast, REGN1908–1909 demonstrated five orders of magnitude higher potency than Cat-SIT-IgG with an IC_50_ of ~580 pM.

In the PCA model, purified Cat-SIT-IgG from three donors, or Non-SIT-IgG, was concentrated and injected into mice to reconstitute the circulating IgG level present in each individual SIT donor. The donors selected represented varying lengths of SIT treatment (13, 20, and 35 months), and included the donor sera that had the highest Fel d 1-specific IgG titer and was most potent in vitro (Donor 10, Supplementary Fig. 4a; Fig. 4a). While Non-SIT IgG did not prevent degranulation, REGN1908–1909 inhibited degranulation as well as or better than purified Cat-SIT-IgG (Fig. 4b). The degree of protection conferred by Cat-SIT-IgG was proportional to the amount of Fel d 1-specific IgG in the individual samples, with the maximum Cat-SIT-IgG protection conferred by Donor 10 which had the highest Cat-SIT-IgG titers supporting the hypothesis that higher titers of polyclonal Cat-SIT-IgG are required for protection. Notably, approximately 5-fold more Fel d 1-specific IgG from Cat-SIT-IgG samples (or 1000-fold more total IgG) was needed per mouse to achieve the same blocking efficacy as REGN1908–1909 [Fel d 1-specific IgG: 0.02 mg/mouse for REGN1908–1909 vs 0.092 mg/mouse for Donor 10 (Fig. 4b, Supplementary Fig. 4b)]. These data show that REGN1908–1909 blocks mast cell degranulation in vivo more efficiently than Cat-SIT patient polyclonal Fel d 1-specific IgG naturally generated after years of SIT and support the concept that the majority of IgG generated by SIT are not functional blockers^14^.

### REGN1908–1909 reduces symptoms in patients with cat allergy

**Fig. 5:**
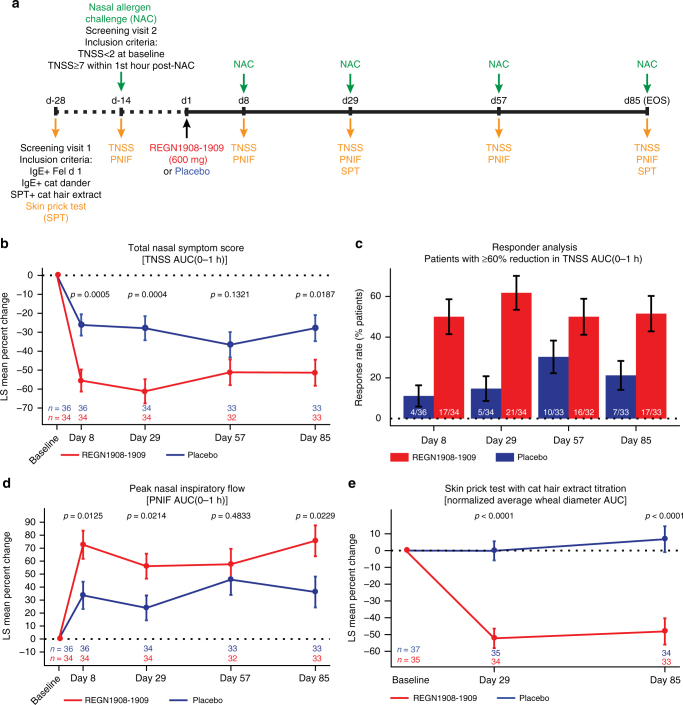
REGN1908–1909 blocks the early phase allergic response to cat extract in patients with cat allergy. **a** Schematic of the study design. **b** Percent change from baseline in total nasal symptom score (TNSS) AUC(0–1 h); LS mean ± SE is shown for the full analysis set (FAS). **c** Percentage of patients with ≥60% reduction in TNSS AUC(0–1 h) is shown at each study timepoint. Numbers on the bars indicate the number of patients represented out of the patient number measured at each timepoint. Percentage of patients ± SE is shown for the FAS. **d** Percent change from baseline in peak nasal inspiratory flow (PNIF) AUC(0–1 h); LS mean ± SE is shown for the FAS. **e** Percent change from baseline in normalized average wheal diameter AUC is shown from the titrated (100-33,000SQU) cat hair extract skin prick test read 15 min after administration on study days 29 and 85. The skin prick test was not administered on study day 8 or day 57 as per protocol. LS mean ± SE is shown for the safety analysis set. Analyses are based on ANCOVA model with treatment group as a factor and baseline as a covariate. For secondary efficacy and exploratory endpoints no control for multiplicity was performed, therefore *p* values for all panels are considered nominal

To determine whether the preclinical protection afforded by REGN1908–1909 could translate into clinical efficacy, we conducted a phase 1b, randomized, double-blind, placebo-controlled proof-of-mechanism study to evaluate if a single subcutaneous (SC) dose of REGN1908–1909 (600 mg; 1:1 ratio) could inhibit cat hair extract-induced allergic symptoms in patients with cat allergy (Fig. 5a). A nasal allergen challenge (NAC) with cat hair extract was selected for proof of mechanism as a reliable, reproducible, and controlled method of measuring allergy symptoms^23^ (Supplementary Fig. 6). NAC studies have been used to test the onset of action and duration of efficacy of intranasal steroids^24^, antihistamines^25^, omalizumab^26^, and subcutaneous immunotherapy (SCIT) approaches^10,27^. Intranasal instillation of allergen causes local allergic symptoms, such as nasal congestion, itching, sneezing, and rhinorrhea, which are associated with IgE-mediated mast cell and basophil degranulation and peak within the EPR^23^. Therefore, the primary efficacy analysis was change in total nasal symptom score (TNSS) area under the curve (AUC) over the first hour after a NAC [TNSS AUC(0–1 h)] from pretreatment challenge to day 8 challenge.

Seventy-three patients were randomized and received the intended study drug (placebo = 37 patients; REGN1908–1909 = 36 patients) (Supplementary Fig. 7). Baseline characteristics were generally balanced between groups, and the majority of patients were polyallergic (Supplementary Table 2). Sixty-eight patients completed the study (34 per group); four patients were removed from the study due to non-compliance of a study site and one patient in the treatment group withdrew consent.

The change in clinical symptoms [TNSS AUC(0–1 h)] from baseline to day 8 NAC (the pre-specified primary endpoint of the study, and the first timepoint tested) was significantly reduced in patients receiving a single SC dose of REGN1908–1909 (600 mg) compared to placebo (ANCOVA, LS mean difference −1.771, *p* = 0.0003; 95% CI[−2.704,−0.838]) (Table 1). Sensitivity analyses of the primary endpoint showed a similar result (Supplementary Table 3). No effect on clinical endpoints was due to an individual study site (*p* = 0.5518). Neither baseline Cat-dander IgE nor Fel d 1 IgE titers correlated with percent change from baseline in TNSS AUC(0–1 h) (*p* = 0.4492, *p* = 0.8157, respectively). Although the NAC model does not robustly induce a LPR^23^, TNSS AUC(1–8 h) was reduced in REGN1908–1909-treated patients compared to placebo at days 8 and 29 (ANCOVA, *p* = 0.0094, and *p* = 0.0074, respectively) (Supplementary Table 4). Component analysis of TNSS revealed that REGN1908–1909 had the greatest impact on two of TNSS components, “Nasal Congestion” and “Rhinorrhea”, which contributed to 34% and 39% improvement on the primary endpoint total score, respectively (Supplementary Table 5).

**Table 1:** Total nasal symptom score (TNSS) primary and secondary efficacy endpoints

	Placebo (*n* = 36)	REGN1908–1909 (*n* = 34)	*p* value
**Primary endpoint: absolute change from baseline to day 8 in TNSS AUC(0–1)**			
Pretreatment mean (SD)	5.660 (1.206)	5.708 (1.436)	0.0003
Day 8 mean (SD)	4.155 (1.889)	2.395 (2.034)	
Change from baseline LS mean (SE)	−1.523 (0.326)	−3.394 (0.335)	
LS mean difference vs placebo (SE)		−1.771 (0.468)	
95% CI		(−2.704, −0.838)	
**Secondary endpoints: percent change from baseline to day 8, 29, 57, and 85 in TNSS AUC(0–1)**			
% change from baseline to day 8 LS mean (SE)	−26.156 (5.635)	−55.503 (5.798)	0.0005
LS mean difference vs placebo (SE)		−29.347 (8.086)	
95% CI		(−45.486, −13.208)	
% change from baseline to day 29 LS mean (SE)	−27.886 (6.36)	−61. 175 (6.360)	0.0004
LS mean difference vs placebo (SE)		−33.289 (8.995)	
95% CI		(−51.253, −15.325)	
% change from baseline to day 57 LS mean (SE)	−36.617 (6.678)	−51.141 (6.781)	0.1321
LS mean difference vs placebo (SE)		−14.524 (9.519)	
95% CI		(−33.551, 4.504)	
% change from baseline to day 85 LS mean (SE)	−27.851 (6.877)	−51.344 (6.877)	0.0187
LS mean difference vs placebo (SE)		−23.492 (9.729)	
95% CI		(−42.935, −4.050)	

Analyses are based on ANCOVA model with treatment group as a factor and baseline as a covariate. For secondary efficacy endpoints no control for multiplicity was performed; therefore, *p* values are considered nominal. Absolute change in TNSS from baseline to days 29, 57, and 85 shown in Supplementary Table 7

*NAC* nasal allergen challenge, *TNSS* total nasal symptom score, *AUC(0–1* *h)* area under the curve (hour 0–1), *SD* standard deviation; *LS* mean least squares mean, *SE* standard error of the LS Mean, *CI* confidence interval

The effect of REGN1908–1909 was clearly maintained for at least a month after the first dose, with evidence suggesting efficacy throughout the 85-day study. REGN1908–1909 treatment led to sustained and clinically meaningful improvement in symptoms (commonly defined as >20% reduction in total symptom score compared to placebo)^28^. REGN1908–1909 treatment reduced TNSS AUC(0–1 h) by 56%, 61%, 51%, and 51% on days 8, 29, 57, and 85 respectively; resulting 29%, 33%, 14,%, and 23% reduction vs placebo and achieving nominal statistical significance vs placebo at every timepoint except day 57 (ANCOVA, *p* = 0.0005, 0.0004, 0.1321, 0.0187, respectively) (Table 1, Fig. 5b). In addition, responder analysis showed that at least 50% of patients receiving REGN1908–1909 achieved a >60% response at every timepoint (Fig. 5c) and only four patients receiving REGN1908–1909 did not achieve >30% response at any timepoint. The duration and magnitude of the subjective TNSS results were reflected in an objective measure of nasal congestion, PNIF, with 39% improvement at day 8 and day 85 (Fig. 5d, Supplementary Table 6). REGN1908–1909 treatment also reduced the acute hypersensitivity response to cat hair extract delivered to a second tissue, the skin, in a titrated skin prick test (Cat-SPT) (Fig. 5e, Supplementary Fig. 8). This skin test is much less subjective than patient-reported allergic symptomology, as reflected by the lack of a placebo response, and may be the best pharmacodynamic measure of the blockade of IgE-driven response. In contrast to patients receiving placebo, where no change in wheal diameter was observed, REGN1908–1909 treatment resulted in a 52% reduction compared to baseline at day 29 (the earliest timepoint tested), with the magnitude of the effect sustained until day 85.

REGN1908–1909 was well tolerated. A total of 107 treatment-emergent adverse events (TEAEs) were observed and were generally balanced between groups (Supplementary Table 7). Two TEAEs were categorized as serious, but determined to be unrelated to treatment by investigator assessment (1 appendicitis in the placebo group, and 1 pyelonephritis in the REGN1908–1909 group). At least one TEAE was observed in 23 (63.9%) patients in the REGN1908–1909 group and 23 (62.2%) in the placebo group. The most frequent TEAE was headache in both groups (six in the REGN1908–1909 group; five in the placebo group). Two mild injection site reactions were observed, one in each group. No subject discontinued due to TEAE, and there were no deaths in the study.

Taken together, these data support that REGN1908–1909 suppresses the early phase allergic response in both mice and humans by binding to cat allergen and preventing IgE:FcεR1 crosslinking and subsequent effector cell degranulation. Furthermore, the similar magnitude of efficacy in all three measures (TNSS, PNIF, and Cat-SPT) observed on day 8, when total antibody drug concentration was >75 mg/L (*C*_max_), and at day 85, when levels were ~11 mg/L, supports that low levels of potent blocking antibodies may prevent allergen-induced early phase allergic response.

## Discussion

In this report, we demonstrated proof of principle that direct administration of allergen-specific monoclonal antibodies provides a well-tolerated, rapid, and effective approach to reducing allergic symptoms. Using Fel d 1, the dominant cat allergen, we systematically demonstrated that the combination of two high-affinity, non-competing Fel d 1-specific IgG4^P^ monoclonal antibodies, REGN1908 and REGN1909, block Fel d 1 binding to cat-allergic polyclonal IgE in vitro and prevents mast cell degranulation in vivo, more efficiently than IgG purified from patients who underwent successful Cat-SIT. This preclinical approach translated to clinical response in an allergy model system. In our study, a single SC dose of REGN1908–1909 in patients with cat allergy resulted in a rapid and clinically meaningful reduction in total nasal symptoms by blocking the early allergic response to nasal challenge with cat allergen.

SIT has been used for over one hundred years^29^, but the use of heterogenous allergen extracts in conventional SIT presents a double-edged sword for patients—in some patients, SIT can cause a reduction in allergic symptoms, but it often requires frequent injections (up to several times a week) over many years, and carries the risk of inducing allergic reactions to the injections^30^. Improvements to SIT have focused on safer and more convenient forms using different routes of administration (e.g. sublingual immunotherapy (SLIT)) to reduce the dose-limiting IgE-mediated side effects of SIT.

New approaches have attempted to recapitulate the protective efficacy of SIT using minimally protective components by focusing on either cellular or humoral responses, while avoiding IgE-mediated side effects^31^. Allergen T-cell peptide vaccines tested the hypothesis that efficacy of SIT was mainly due to modulation of T-cell responses^32^. This approach, advanced by Circassia Pharmaceuticals, identified and administered short allergen peptides that correspond to major T-cell epitopes, but do not bind IgE or induce an IgE or IgG humoral response^33–35^. However, in a phase 3 study of cat allergy and a phase 2b study in house dust mite allergy^36^, this approach failed to demonstrate significant benefit^8^. Approaches testing the hypothesis that SIT efficacy is driven by humoral responses are also in clinical development, focusing on generating high titers of blocking IgGs either with peptide vaccines or our approach of passive administration of monoclonal antibodies, as described in this study. Peptide vaccines using long, continuous overlapping peptides comprising the whole allergen sequence^37^ or longer B-cell peptide fusion proteins to induce IgG^38^ have demonstrated clinical efficacy in birch^37^ and grass^39^ allergy, respectively. These peptide immunization approaches have the advantage of administering multiple peptides in a given preparation. By design, peptide vaccine approaches discourage the formation of conformational structure to prevent IgE crosslinking upon administration; and, in the case of B-cell fusion peptide vaccines, substantial efforts have gone into identifying and/or engineering non-allergenic peptides that result in functional blocking IgG, including for Fel d 1^40^. In contrast to peptide vaccines, our approach allows selection of the most potent combination of blocking antibodies generated in response to whole allergens naturally and agnostically by genetically humanized VelocImmune mice^17,18^. Interestingly, REGN1908 and REGN1909, the two most potent blocking antibodies, bind to conformational Fel d 1 epitopes (Fig. 2b, c). Furthermore, passive administration enables direct delivery of high, uniform concentrations of two blocking antibodies (>75 mg/L total) with a single dose, avoiding the need for repeated allergen or peptide administrations, adjuvants, and subsequent patient immune response to generate high antibody titers. Taken together, passive immunotherapy with monoclonal antibodies may be the most efficient approach to obtaining high titers of quality allergen-blocking IgG.

We employed a controlled nasal challenge model to explore the mechanistic question of whether passive administration of anti-allergen monoclonal antibodies can block allergen binding to IgE in patients with cat allergy, thereby reducing allergic symptoms. Our data support that a pair of high-quality anti-allergen-blocking antibodies is sufficient to reduce allergic symptoms in response to nasal provocation^41^, supporting the hypothesis that the generation of allergen-specific blocking IgG is the key mechanism of SIT^42^. Few well-controlled, long-term studies have specifically examined efficacy of SIT in cat allergy; however, a 3-year study examining the effects of 2 years of grass SCIT and SLIT with 1 year of therapy discontinuation on acute allergic response using a similar NAC design and TNSS endpoint has been published^43^. In this study, the highest mean titers of allergen-specific IgG4 were observed at the end of 2 years of SCIT therapy, the timepoint at which the greatest reduction in TNSS was also observed. Although allergen-specific IgG4 levels tracked with efficacy, there was high interpatient variability (2–61 mg/L; median = 18.9 mg/L)^43^ and presumably only a proportion of these anti-allergen antibodies are functional blockers^14^. Furthermore, efficacy was lost one year after cessation of therapy, as allergen-specific IgG4 levels also declined^43^. In our clinical study, which used similar NAC methodology, but with a perennial allergen, direct administration of high doses of blocking IgG4 can be achieved rapidly, resulting in a magnitude of acute symptom relief similar to 2 years of SCIT, and greater than that observed 1 year after treatment cessation, without patients undergoing 2 years of immunotherapy^43^. In addition, the improvement observed in this study may be 8-fold more effective than targeting downstream mediators, such as antihistamines, and 4-fold more effective than nasal steroids^44^. Future studies will investigate this mechanism on clinical efficacy in real-world exposure settings.

Several studies report that an increase in IgG/IgE ratio is an important component of SIT desensitization^9,11,12^ and a protective feature of natural allergen tolerance^45^. In our study, direct administration of REGN1908–1909 resulted in total serum antibody concentrations >75 mg/L at day 8 post-injection and >11 mg/L throughout the study. While IgE levels were unable to be measured during treatment, the highest baseline anti-Fel d 1 IgE titer for a cat-allergic subject enrolled in our study was 76.8 kU/L, or ~184 ug/L. Therefore, it is assumed that REGN1908–1909 is in vast excess of circulating anti-Fel d 1 IgE, though tissue levels of either allergen-specific IgE or IgG are unknown. Since the key to preventing initiation of the allergic cascade is to block allergen binding to cell-bound IgE:FcεR in tissue, a clue that REGN1908–1909 may also be in excess of IgE in tissue comes from the skin prick test results. REGN1908–1909 reduced the wheal response by ~50% at the first study day tested (day 29) and maintained this reduction at day 85. Compared to increasing allergen-blocking IgGs, simply blocking IgE binding to IgE receptors (Xolair, anti-IgE, omalizumab^46^) may not be as effective. To impact wheal response in a SPT by reducing IgE alone, omalizumab had to be administered for 8 weeks to observe reduction in wheal^47^, and requires maintenance of free serum IgE levels <10 IU/mL^48^. These data demonstrate that simply augmenting the amount of functional blocking IgG rapidly and efficiently prevents allergen binding to IgE:FcεR. Our confirmation of the critical function of the IgG:IgE ratio in allergy is consistent with clinical investigation of an antibody blocking the IL-4/IL-13 pathway (Dupixent, anti-IL-4Rα, dupilumab), which reduces IgE and has shown impressive efficacy in multiple allergy-related conditions such as asthma^49^, atopic dermatitis^50–52^, and nasal polyposis^53^; blocking this pathway could provide a longer-term approach to the treatment of allergy.

This clinical proof-of-principle study examined the potential of passive immunotherapy with monoclonal antibodies to Fel d 1 to block acute allergic symptoms triggered by nasal provocation with cat extract. The majority of patients receiving REGN1908–1909 experienced symptom amelioration with at least 50% of patients achieving 60% improvement in TNSS at every timepoint, clinically establishing the immunodominance of Fel d 1 as the driver of cat allergy symptoms. While not all patients achieved a clinical response, similar interpatient variability was observed preclinically in a functional ex vivo basophil assay: 80% inhibition of basophil activation was observed in 7/10 donors (Fig. 2e). The variability observed both preclinically and clinically suggests either that REGN1908–1909 does not block Fel d 1 binding to a proportion of patients’ IgE repertoire or that Fel d 1 is not the main driver of cat-allergic symptoms for some patients. However, unique to a passive immunization approach, these data also suggest a potential mechanism to predict patient response to REGN1908–1909 therapy: Since ex vivo BATs have been shown to correlate with nasal response to allergen challenge^54^, it will be interesting to determine whether pre-testing the ability of REGN1908–1909 to block Fel d 1 from crosslinking patient IgE in an ex vivo functional basophil bioassay can target therapy to patients who will receive maximal benefit.

The presumed mechanism of REGN1908–1909 is blockade of allergen binding to IgE:FcεR on mast cells (and other effector cells) by the injection of competing allergen-specific IgG4 antibodies. However, IgG4 antibodies may also decrease T-cell activation by preventing facilitated antigen presentation^55^, decreasing IgE production by preventing B-cell activation^56^, or inhibiting effector cell activation through co-engagement of inhibitory receptors^57,58^. In our studies, we did not examine downstream immunological effects such as T-cell response, nor were we able to measure allergen-specific IgE. Additionally, this approach is not expected to result in immunological memory. The majority of allergic individuals are polyallergic; however, in polysensitized individuals the combined allergen load may act additively to exceed the threshold of effector cell activation causing increased responsiveness to lesser amounts of individual allergens^59,60^. Therefore, it would be interesting to determine whether preventing mast cell degranulation and allergen presentation over time could result in an overall shift toward a less reactive T-cell response, or alter the underlying type 2 inflammation causing allergic disease even in polyallergic individuals.

The most consistent immunological response associated with desensitization across SIT studies is increased allergen-specific IgG4 levels^14^. Therefore, passive administration of anti-allergen-blocking antibodies has the potential to work in a variety of allergic settings considering three main parameters: allergen size, allergen diversity, and number of immunogenic epitopes per allergen. Fel d 1 is the main driver of cat allergy and has multiple immunogenic epitopes^61^, but is small in size, 18 kDa. Together these characteristics enabled the selection of two antibodies which provided acute symptomatic relief for the majority of patients. For other allergens, more than two antibodies may be required, depending on the number of immunogenic epitopes and their spatial orientation. For example, larger allergenic proteins may have multiple, spatially distributed immunogenic epitopes, such as Cry J 1 from Japanese Cedar (40 kDa)^62^, or Phl p 5a^63^. For allergies driven by more than one allergenic protein (e.g. peanut allergy), advances in diagnostics such as component resolved diagnosis^64^ or ex vivo functional basophil assays can reveal the dominant proteins driving patient reactivity and enable selection of the appropriate antibody combination for a given patient. Thus, a personalized medicine approach to allergy may require generation of a repertoire of anti-allergen blocking antibodies.

With REGN1908–1909, we have demonstrated proof of principle for rapid, effective, and potentially long-lasting reduction in cat allergy symptoms by directly targeting Fel d 1, the major cat allergen. In addition, REGN1908–1909 was well tolerated, with no anaphylaxis or evidence of hypersensitivity observed. The clinical response in this allergy model system validates a new paradigm for developing anti-allergen antibodies for treating allergic diseases: Preclinical selection of potent, fully human anti-allergen antibodies that block allergen binding to a majority of allergen-specific IgE, directly administered to patients for rapid and meaningful symptom relief. Applying this innovative, systematic approach to other allergens may enable development of a repertoire of anti-allergen therapeutics for personalized allergy management.

## Methods

### Natural and recombinant Fel d 1

Both natural Fel d 1 (nFel d 1; Indoor Biotech, Catalog #LTN-FD-1) and recombinant Fel d 1 (rFel d 1) were used in in vitro assays. Recombinant Fel d 1 was produced following the design of Kaiser et al.^65^ who showed that single-chain fusions were structurally and functionally equivalent to the natural Fel d 1 heterodimer. The Regeneron-produced recombinant proteins include amino acids 18–109 of Fel d 1 Chain 2 (NP_001041619.1) at the N-terminus fused directly in-line to amino acids 23–92 of Fel d 1 Chain 1 (NP_001041618.1) with a D27G mutation and a C-term myc–myc–hexahistidine (mmh) tag. The proteins were produced in Chinese Hamster Ovary (CHO) cells with either a monomeric (mmh) or a dimeric ((mouse IgG2a Fc (mFc)) C-terminal tag (rFel d 1.mmh and rFel d 1.mFc, respectively). In addition, FcεR1α (the high affinity receptor for IgE) extracellular domain protein was produced as a dimeric Fc fusion with a C-terminal mouse Fc tag (hFcεR1α.mFc) to support development of the ELISA-based competition assay.

### Generation of REGN1908 and REGN1909

REGN1908 and REGN1909 are fully human monoclonal antibodies to Fel d 1 produced with IgG4^P^ isotype Fc domains. The IgG4 constant domain contains a serine to proline amino acid substitution (S228P, EU numbering) in the hinge region that reconstructs the human IgG1 hinge sequence (CPPC) to promote stabilization of disulfide bonds between the two heavy chains^19^, therefore designated IgG4^P^. Briefly, VelocImmune mice^17,18^ were immunized with recombinant dimeric Fel d 1 (rFeld1.mFc). Hybridomas producing REGN1908 and REGN1909 were isolated and the variable regions were cloned onto plasmid vectors containing kappa light chain constant regions and hIgG4^P^ heavy chain constant regions, respectively. The selected antibodies were produced in Regeneron’s proprietary cell line cloned from CHO cells. CHO-K1 cells were authenticated by isozyme testing according to ICH guidelines and have been tested for mycoplasma contamination. Lead antibodies were selected based on affinity and the ability to bind non-competitively to Fel d 1.

### Isolation and concentration of IgG from patient sera

Serum from patients with cat allergy who underwent physician determined successful immunotherapy (Cat-SIT) and cat-allergic control patients (Non-SIT) was purchased from Dr. Jonathan Corren. Uses of human material were approved by Schulman Associates IRB. Cat allergy was physician diagnosed by allergy skin prick test and clinical symptoms. All patients with cat allergy were polyallergic. Immunotherapy was performed using cat extract diluted with human serum albumin in the absence of adjuvants with treatment success determined by the physician. Patient serum was separated from donor blood (purchased from Dr. Jonathan Corren) by centrifugation at 3000 rpm, passed through 0.22 μm filter, incubated overnight with 50 mL of Protein G sepharose beads at 4 °C, then poured into column and SIT IgG and eluted with Pierce IgG elution buffer. After neutralization of the eluate with 1 M Tris, pH 8.5, samples were dialyzed against PBS, pH 7.2. All samples were concentrated approximately 10-fold using an Amicon Ultracel with a 50 k MW cut off.

### Quantitation of IgG from patient sera

Total and Fel d 1-specific IgG levels were quantitated in sera samples from human patients undergoing SIT therapy using a standard ELISA. Ninety-six-well microtiter plates (Thermo Scientific) were coated with 2 µg/mL of either natural Fel d 1 (LoTox Indoor Biotechnologies) or anti-human IgG (Jackson Immunoresearch) in phosphate-buffered saline (PBS, Irvine Scientific) overnight at 4 °C. The next day, plates were washed with PBS containing 0.05% Tween 20 (PBS-T, Sigma-Aldrich) four times using a plate washer (Molecular Devices). Plates were then blocked by incubation for 1 h at room temperature (RT) with 250 µL of 0.5% bovine serum albumin (BSA, Sigma-Aldrich) in PBS. Sera or purified IgG from human patients undergoing SIT were serially diluted threefold in 0.5% BSA-PBS starting at 1:1000 (for Fel d 1 specific IgG) and 1:27,000 (for total IgG), added to the blocked plates in duplicate, and then incubated for 1 h at RT. The last two wells were left blank to be used as a secondary antibody alone control (background control). For total IgG quantitation, a standard curve was generated using human IgG (Thermo Scientific, # 31,154) starting at 1 µg/mL and diluted 3-fold across the plate. For Fel d 1-specific IgG quantitation, a standard curve was generated using an anti-Fel d 1 monoclonal antibody (H4H1238N) also starting at 1 µg/mL and diluted 3-fold across the plate. Goat anti-human IgG-Fc-Horse Radish Peroxidase (HRP) conjugated secondary antibody (Jackson Immunoresearch) was then added to the plates at 1:5000 dilution and incubated for 1 h at RT. Plates were washed with PBS-T in between each step of the protocol. To develop the colorimetric reaction, TMB/H_2_O_2_ substrate was added to the plates and incubated for 20 min. The reaction was stopped using 2 N sulfuric acid (H_2_SO_4_, VWR). Absorbance was subsequently measured on a spectrophotometer (Victor, Perkin Elmer) at 450 nm. Total and Fel d 1 specific IgG were computed from the respective standard curve plots using the Graphpad PRISM software.

### Cat-SIT IgG binding studies

Cat-SIT-IgG binding studies were performed on Octet HTX biosensor and all the samples were prepared in a buffer containing 10 mM HEPES, 500 mM NaCl, 1 mg/mL BSA, 0.02% sodium azide, and 0.05% surfactant Tween-20 with pH 7.4 (HBS-BT). HIS1K Octet biosensors were first dipped in wells containing 2 µg/mL of rFel d 1.mmh for 60 s to capture 0.05 nm of rFel d 1.mmh followed by dipping Octet biosensors in wells containing 100 nM of concentrated Fel d 1-specific IgG present in different Protein G-purified Cat-SIT IgG samples. The specific binding sensorgrams were generated by a double referencing procedure by subtracting any interaction of Cat-SIT IgG over the reference surface (blank HIS1K Octet biosensor) from the Cat-SIT IgG binding to the Fel d 1.mmh captured surface; thereby removing any observed non-specific binding signal. In addition, rFel d 1.mmh captured biosensors were dipped in HBS-BT buffer to allow subtraction of signal changes resulting from the natural dissociation of captured rFel d 1.mmh from the HIS1K biosensor. Octet binding data were double reference subtracted and binding kinetic parameters were measured by fitting the data to a 1:1 binding model with mass transport limitation using Scrubber 2.0c.

### SPR binding studies

Binding kinetics studies for REGN1908 and REGN1909 were performed on Biacore T200 using 10 mM HEPES, 150 mM NaCl, 3 mM MgCl_2_ + 3 mM CaCl_2_, 0.05% (v/v) surfactant P20, pH 7.4 (HBSP+ +) as running buffer at a flow rate of 50 μL/min. Around 94–163 RU of REGN1908 or REGN1909 was first captured on different flow cells using the goat anti-human Fcγ coupled sensor surface. Different concentrations of nFel d 1 or rFel d 1.mmh, serially diluted by 2-fold in HBSP+ + buffer were later injected over the antibody captured surfaces for 2.5 min followed by 15 min dissociation phase. Specific Biacore kinetic sensorgrams were obtained by a double referencing procedure by first subtracting any interaction of nFel d 1 or rFel d 1.mmh over the reference surface (goat anti-human Fcγ coupled surface only) from the nFel d 1 or rFel d 1.mmh binding signal to REGN1908 or REGN1909 captured surfaces; thereby removing any refractive index changes. In addition, injections of HBSP+ + buffer were performed to allow subtraction of RU signal changes resulting from the natural dissociation of captured REGN1908 or REGN1909 from the goat anti-human Fcγ coupled surface. The binding kinetic parameters were obtained by globally fitting the double reference subtracted data to a 1:1 binding model with mass transport limitation using the Biacore T200 Evaluation Software, version 1.0.

Sequential binding competition studies were performed on Biacore T200 by immobilizing 4682–6683 RU of REGN1908 and REGN1909 over separate flow cells of a CM5 sensor chip using standard amine-coupling chemistry reported earlier. Freshly prepared and degassed HBS-P buffer (10 mM HEPES, 150 mM NaCl, 0.05% surfactant P20), pH7.4 was used as running buffer during the entire coupling steps. The coupled chip surface was then washed and treated with 10 mM glycine-HCl, pH 1.5 to remove uncoupled residual proteins. 200 nM nFel d 1 was first injected over the sensor surface coupled with REGN1908 or REGN1909 for 10 min followed by individual injections of REGN1908 and REGN1909 (25 μg/mL). The entire experiment was performed using HBS-P+ + running buffer at a flow rate of 10 μL/min.

### Epitope determination by hydrogen/deuterium exchange

In order to determine the epitopes of Fel d 1 recognized by REGN1908 and REGN1909, HDX studies were performed for each antibody co-complexed with rFel d 1.mmh. Amide protons on recombinant Fel d 1 (rFel d 1.mmh) were first exchanged in D_2_O, then the deuterated rFel d 1.mmh was complexed with either REGN1908 or REGN1909 prior to the back-exchange in H_2_O. The control experiment is that the complexed rFel d 1.mmh with either antibody was deuterated in D_2_O and back-exchanged in H_2_O. The solution was then quenched in cold (4 °C) acidic (pH 2.5) aqueous solution to minimize back-exchange and subjected to proteolysis and mass spectrometry analysis. Fel d 1-derived peptic peptides that exhibited increased mass (very likely due to retained deuterons from the antibody protection) greater than 0.2 mass units relative to control experiment were defined as the binding epitopes based on H/DX methodology.

### REGN1909 binding to Fel d 1 by X-ray crystallography

A Fab fragment of REGN1909 was cloned and expressed in HEK293 cells, then purified by Protein A affinity chromatography. Recombinant Fel d 1 (chain 2(N50A)–chain 1(G31D), with a C-terminal myc–myc–His6 affinity tag) was purified as described above. rFel d 1.mmh and REGN1909 Fab were mixed in approximately 1:1 molar ratio, incubated at RT for 1 h, then the complex was separated from a slight excess of Fab by size exclusion chromatography. rFel d 1–Fab complex was concentrated to 20 mg/ml in HEPES-buffered saline, then crystallized against a reservoir solution of 0.2 M calcium acetate, 0.1 M sodium cacodylate pH 6.5, 40% (v/v) polyethylene glycol 300. Crystals were harvested directly from the mother liquor and frozen in liquid nitrogen.

Diffraction data to 2.9 Å were collected on a rFel d 1-Fab crystal at beamline 5.0.2 of the Advanced Light Source, Lawrence Berkeley National Laboratory. The structure was solved by molecular replacement using Phaser^66^, with PDB code 1PUO^65^ as the search model for the Fel d 1 component, and PDB code 2R8S^67^ for the Fab component. The structure was refined using refmac5, and rebuilt using coot^68^. Coordinates for the final rFel d 1-Fab structure have been deposited in the RCSB Protein Data Bank with accession code 5VYF. See Supplementary Table 9 for data and refinement statistics.

### Allergen-specific blocking ELISA

The ability of anti-Fel d 1 monoclonal antibodies or purified IgG from SIT patient serum to block Fel d 1 binding to plate-captured IgE from allergic human donor plasma/sera was determined using a blocking ELISA. Microtiter plates were coated overnight at 4 °C with human FcεR1α (the high affinity receptor for IgE) extracellular domain protein with a C-terminal mouse Fc tag (hFcεR1a.mFc). Plates were blocked with 0.5% BSA (w/v) for 1 h at RT. Plasma from allergic donors was diluted 5-fold and total IgE was captured over the receptor-coated surface. A constant amount of recombinant Fel d 1.mmh (0.7 nM) was pre-mixed with serial dilutions of anti-Fel d 1 monoclonal antibodies and Fel d 1 specific SIT IgG starting from 10 µg/ml each in 3-fold serial dilution and incubated for 1 h at RT to allow Fel d 1–antibody interaction to reach equilibrium. The antibody–Fel d 1 mixture was then added to the IgE-coated plate for 1 h. Plates were subsequently washed and the amount of free Fel d 1.mmh bound to plate was detected using an anti-myc antibody (clone 9E10 produced in-house as a human IgG1 isotype) conjugated to HRP, and incubated at a 1:10,000 dilution, by incubating for 1 h at RT. Plates were washed with PBS-T between each step of the protocol. To develop the colorimetric reaction, TMB/H_2_O_2_ substrate was added to the plates and incubated for 20 min at RT. The reaction was stopped using 2 N sulfuric acid (H_2_SO_4_; VWR, #BDH3500-1). Absorbance was subsequently measured on a spectrophotometer (Victor, Perkin Elmer) at 450 nm. The concentration of antibody required to inhibit the signal of a constant concentration of Fel d 1 by 50% (IC_50_) was determined using the Prism software.

### Basophil activation assessed by phosphorylated Erk

Blood drawn from patients with cat allergy (*n* = 9 patients, 10 total samples based on one repeat visit) was shipped at RT for same-day delivery (Bioreclamation IVT). PBMCs were purified by centrifugation on a Ficoll layer, washed three times in pre-warmed RPMI media (Gibco), resuspended in pre-warmed serum-free X-Vivo 15 media (Lonza) and plated on a 96-well plate (as single points). The cells were then incubated at 37 °C for 30 min. In parallel, a 2× stimulation plate was prepared that included a dose response of purified nFel d 1 as well as dose responses of anti-Fel d 1 antibodies (2.56 fM-200 nM) mixed with a constant dose (final concentration 200 pM) of purified natural Fel d 1 (Indoor Biotechnologies). The stimulation plate was also incubated for 30 min at 37 °C. The cells were then stimulated at 37 °C using a 96-well multichannel pipette, and the stimulation was stopped after precisely 5 min by adding 1 volume of pre-warmed Cytofix (BD). After 15 min of fixation, the cells were washed twice in MACS buffer and made permeable by resuspending and storing them in ice-cold methanol overnight at −20 degrees. The cells were then washed three times with MACS buffer, resuspended for 10 min in human Fc blocker (ebioscience) and then stained with an antibody cocktail containing pErk-Alexa 488 (Cell Signaling, Catalog #13214s, clone 197G2), CD123-BUV395 (BD, Catalog #564195,clone 7G3), and HLA-DR-APC (BD Catalog #559866, clone G46-6) antibodies for 30 min. Cells were washed twice with MACS buffer, incubated for 15 min with Cytofix (BD) diluted 1:4 in PBS to fix the stain, resuspended in MACS buffer and acquired in an LSR-Fortessa instrument. Data were analyzed by calculating the median fluorescence intensity (MFI) of phosphorylated Erk staining within the basophil gate. Percent Max Inhibition was calculated as

$$100-(({\mathrm{100}} \times {\mathrm{Maximum}}\,{\mathrm{Antibody}}\,{\mathrm{Response}})/{\mathrm{Isotype}}\,{\mathrm{Response}})$$

Maximum antibody response was the average MFI of phosphorylated Erk in the top three doses of antibody in the dose–response curve (plateau of the curve) minus the baseline MFI (average of replicate unstimulated samples), and isotype response is the average of all the MFI values in the dose response of a Regeneron-produced IgG4^P^ isotype control antibody minus the baseline MFI.

### Basophil activation assessed by CD203/CD63 in cat allergy patients

To evaluate antibody-mediated inhibition of basophil activation, a 20 pM final constant concentration of Fel d 1 (Indoor Biotechnologies) was pre-incubated for 30 min at 37 °C with REGN1908–1909 combination or IgG4^P^ isotype control antibody at final concentrations ranging from 0.8 fM to 1.0 μM. Concurrently with the Fel d 1 and antibody pre-incubation, PBMCs (BioreclamationIVT) were purified from fresh whole blood from allergic donors by Ficoll density gradient centrifugation. The purified PBMCs were washed, resuspended in X-VIVO 15 media (Lonza), and plated in duplicate columns in a v-bottom, polypropylene, 96-well plate (approximately 5 × 10^5^ cells/well). To prime the basophils contained in the overall PBMC population for activation, hIL-3 (R&D Systems, 0.3 nM) was added to the cell suspension and the plate was incubated at 37 °C for 10 min. The pre-incubated antibodies and Fel d 1 were then added to the primed PBMC. Cells without the addition of antibody were included as negative control samples and a dose response of Fel d 1 ranging from final concentrations of 7.8 aM to 10.0 nM was included as a positive control. The cells were then incubated at 37 °C for 20 min to facilitate basophil activation. Activation was subsequently stopped by incubation at 4 °C for 5 min. Basophil activation was then evaluated by flow cytometry. The cells were stained at 4 °C for 20 min with either an antibody cocktail containing anti-HLA-DR-FITC (Beckman Coulter, Catalog #IM0463U, clone B8.12.2), anti-CD123-APC (BD, clone 7G3, Catalog #560087), and anti-CD203c-PE (Beckman Coulter, Catalog #IM3575, clone 97A6) or a cocktail containing anti-HLA-DR-PE-Cy7 (Biolegend, Catalog #307616, clone L243), anti-CD123-APC (BD, Catalog #560087,clone 7G3), and anti-CD63-FITC (Beckman Coulter Catalog #IM1165U clone CLBGran/12). After staining, the cells were washed in a 1:20 dilution of MACS BSA Stock Solution (Miltenyi Biotec) in autoMACS Rinsing Solution (Miltenyi Biotec), fixed in Cytofix (BD) diluted 1:4 in Dulbecco’s phosphate-buffered saline (Gibco), and analyzed on a flow cytometer (BD LSRFortessa X-20). The gating strategy (Supplementary Fig. 6c) was used to identify basophils within the larger population of PBMC and to determine levels of basophil activation. Samples were run to collect approximately 1000 events determined to be basophils. Basophils were defined as singlet, lymphoid, HLA-DR-, and CD123+. Activated basophils were further defined as CD203c^Hi^ or CD63^Hi^. To specify a baseline level of activation, gates were set so that 10% of basophil events from hIL-3-primed, unstimulated samples (no Fel d 1) were positive for activation (CD203c^Hi^ or CD63^hi^). These gates were then applied to all other experimental conditions to determine the relative level of basophil activation.

### Mouse experiments

Female, 7, 8 week old Balb/c mice from Jackson Laboratories were used for all mouse studies.

For the entire duration of the experiment, animals remained housed in the Regeneron animal facility under standard conditions, and were allowed to acclimate for at least 7 days prior to being placed on study. All animal experiments were performed in accordance with the guidelines for the Institutional Animal Care and Use Committee at Regeneron. For preclinical mouse studies, no statistical methods were used to predetermine sample size. Mice were randomly assigned to treatment groups without predefined criteria and blinding was not able to be performed due to obvious color change of the ear.

### Passive cutaneous anaphylaxis mouse model

Antisera used for passive intradermal (ID) administration were previously generated by immunizing Balb/c mice with either Fel d 1, standardized cat hair extract, or crude peanut allergen extract (irrelevant control antisera) using alum adjuvant. Sera from 10 to 20 mice were pooled and used for passive administration of allergen-specific IgE. On day 1, groups of Balb/c mice (exact “n” noted in the figure legend of the corresponding figure) received a SC injection of REGN1908, REGN1909, REGN1908–1909, concentrated Cat-SIT IgG or Non-SIT IgG, or a Regeneron generated IgG4^P^ isotype control antibody. Three days later, antisera generated to Fel d 1 or cat hair extract, or peanut (negative control) was injected ID into the right and left ears, respectively, allowing allergen-specific IgE to bind FcεR on mast cells. Each anti-serum was standardized to contain 1–25 ng IgE per injection (exact concentration noted in respective experiment). Twenty-four hours after local administration of allergen-specific antisera, mice were challenged by intravenous (IV) injection of 0.25–1μg Fel d 1 (exact concentration noted in respective experiment) or 250 Bioequivalent allergy units (BAU) cat hair extract diluted in PBS containing 0.5% Evans blue dye (Sigma, Catalog #E2129). One hour after allergen challenge, mice were sacrificed, Evans blue dye was extracted from ear tissue and spectrophotometrically quantitated using a standard curve. Ears were then dried and weighed.

Data are presented as ng/mg with value obtained from each peanut ear subtracted from the corresponding value for the Fel d 1 challenge ear. Circulating human antibody levels were measured at the day of sacrifice by ELISA. Microtiter plates (VWR, Catalog # 62409-024) were coated overnight at 4 °C with 1 µg/mL goat anti-human IgG Fc (Jackson ImmunoResearch, Catalog #109-005-098), washed four times with a plate washer (Molecular Devices), and blocked with 0.5%BSA (w/v). Two-fold dilutions of mouse serum were added in duplicate wells, and incubated for 1 h at RT. Plates were washed 4 times and bound antibodies detected using goat anti-human IgG Fc HRP (Jackson ImmunoResearch, Catalog #109-035-098) and BD Opt EIA TMB Substrate (BD Pharmingen, Catalog #555214). The reaction was stopped using 2 N sulfuric acid (Sigma) and absorbance at 450 nm read on a spectrophotometer (Molecular Devices).

### Preclinical statistical analysis

Statistical analysis was assessed using GraphPad Prism 6. Analysis of the differences between two groups was assessed using the Student’s paired or Student’s unpaired two-tailed *t* test.

For multiple group animal studies, normality of the data was evaluated using the Shapiro–Wilk if *n* > 7 or the Kolmogorov–Smirnov test if *n* <7. If data passed the normality test, and standard deviations of the different groups were not statistically different from each other as assessed by the Brown–Forsythe test, results were interpreted by one-way analysis of variance followed by the Tukey post hoc test for multiple comparisons. If data failed to pass the normality test, or standard deviations were significantly different, results were interpreted using the Kruskal–Wallis test followed by Dunn’s post hoc test for multiple comparisons. Differences were considered to be statistically significant when *p* < 0.05.

### Clinical study design

R1908–1909-ALG-1325.03 (NCT02127801) was a multicenter phase 1b, randomized, double-blind, placebo-controlled, single SC dose, proof-of-mechanism study conducted in six study centers in Europe and Asia-Pacific. The first subject visit was September 10, 2014 and the last subject completed day 85 on December 15, 2015.

Study participants eligible for randomization were stratified in two blocks: the London site (Quintiles phase I Unit), and all other study sites combined, implemented through a central interactive voice response system. Study participants were randomized 1:1 on day 1 to receive a single SC dose of REGN1908–1909 (600 mg total, 1:1 antibody ratio; *n* = 37) or placebo (*n* = 36). Study participants, the principal investigators, and study site personnel remained blinded to all randomization assignments throughout the study. The Regeneron study director, medical monitor, study monitor, and any other Regeneron and contract research organization personnel who were in regular contact with the study site remained blinded to all subject randomization assignments. A Study Manual was developed to standardize techniques across the sites. Personnel from all sites underwent centralized training on the Manual and all study procedures at Quintiles Unit in London. The number of staff at each site performing specific assessments was limited to minimize the inter-operator variability.

The protocol was approved by the appropriate ethics committees/institutional review boards, and each patient gave written consent at screening visit 1. The study was conducted in compliance with institutional review board regulations, the International Conference on Harmonization Guidelines for Good Clinical Practice, and the Declaration of Helsinki. This study and all uses of human material were approved by the following national competent authorities: Centrale Commissie Mensgebonden Onderzoek (CCMO), Netherlands; Medsafe, New Zealand; Läkemedelsverket Medical Products Agency, Sweden; and Medicines and Healthcare products Regulatory Agency (MHRA), United Kingdom.

### Patient population

Eligible participants were 18–55 years of age with cat-induced allergic rhinitis and cat sensitization confirmed at screening. To confirm cat-sensitization, participants underwent screening at two visits, day −28 and day −14 (±2 days). At screening visit 1, participants were screened for allergen-specific IgE, underwent a skin prick test with cat hair extract (cat-SPT, Aquagen, ALK-Abello) and other allergen extracts, and were tested for lung function (FEV1). Participants were eligible for screening visit 2 based on IgE titers specific for Fel d 1 and cat hair extract >0.35kAU/l each, cat-SPT mean wheal diameter of >3 mm compared to a negative control SPT, and normal lung function. Patients were excluded if they had prior history of SIT or vaccination with cat allergen, or anti-IgE therapy; SIT to other allergens within 3 months prior to screening; or were living with or chronically exposed to a cat.

At screening visit 2, a single NAC was performed using increasing doses of cat hair extracts (100-33,000 SQ-U/ml). Briefly, cat hair extract was applied intranasally every 10 min for 1 h, or until a TNSS >7 was reached. TNSS (measured on a 0–12 scale) is a composite patient symptom assessment of congestion, itching, and rhinorrhea (each graded on 0–3 scale, 3 being severe), and sneezing (3 being >5 sneezes). Cat-sensitized patients were eligible for enrollment based on having a TNSS <2 prior to the screening NAC (time 0), and peak TNSS >7 within one hour of NAC initiation. PNIF (measured in nasal patency, l/min) was also measured at screening visit 2.

Eligible study participants were randomized to receive study drug or placebo on study day 1 (14 days after screening visit 2). On study days 8, 29, 57, and 85, study participants underwent NAC using the same allergen titration required for each individual subject to reach TNSS >7 at their 2nd screening visit, not to exceed the maximum dose established in the screening visit, regardless of whether TNSS>7 was reached. At each study visit, TNSS, and PNIF and were measured pre-NAC, then at 10, 30, and 60 min during the first hour, and once per hour for 8 h to measure the LPR. Serum samples were collected at each study visit, and a repeat cat-SPT was performed on study days 29 and 85.

### Efficacy endpoints

The primary efficacy endpoint was change in TNSS AUC between pretreatment and day 8 NAC over the first hour of challenge (0–1 h, early phase allergic response). Secondary endpoints were percent change in TNSS AUC from pretreatment to day 8 NAC over the first hour; change and percent change in TNSS AUC from pretreatment NAC to days 29, 57, and 85 over the first hour; and change and percent change in TNSS AUC from hours 1 to 8 post-challenge (LPR) from pretreatment NAC to days 8, 29, 57, and 85. Exploratory endpoints included change and percent change from pretreatment NAC to days 8, 29, 57, and 85 in peak TNSS, and peak PNIF; as well as PNIF AUC from 0 to 1 h, and 1 to 8 h. Responder analysis was performed ad hoc. Pharmacokinetic parameters were also measured.

Percentage change from baseline to day 29 or day 85 in mean wheal diameter AUC for the titrated cat hair extract skin prick test (100-33,000 SQ-U/ml) measured 15 min post-application was an additional exploratory endpoint. The test was performed with duplicate serial dilutions of Aquagen (ALK-Abello) cat hair extract using six dose titrations ranging from 100 to 33,000 SQ-U/ml. Single probes with a negative control solution (saline) and a positive histamine control were administered in duplicate simultaneously with cat hair extract probes on the opposite arm. 15 min after application, the wheal diameters were recorded. Mean wheal diameters were calculated by adding the longest diameter to the longest orthogonal diameter and dividing by 2. For each of the duplicate skin prick tests, the longest and longest orthogonal diameters should be recorded, and the mean diameter of each wheal calculated to two decimal places. The mean wheal diameters from the duplicate skin pricks were then averaged.

For the titrated cat skin prick test, the formula used to calculate the normalized average wheal diameter AUC was

$$\begin{array}{l}\left[{{(t1-t0)}}\left( {{{D1}} + {{D0}}} \right)/{{2}} + {{(t2 - t1)}}({{D2}} + {{D1}})/{{2}} + {{(t3 - t2)}}({{D3}} + {{D2}})/{{2}}\right.\\ \left.+ {{(t4 - t3)}}({{D4}} + {{D3}})/{{2}} + {{(t5 - t4)}}({{D5}} + {{D4}})/2\right]/{{(t5}} - {{t0)}}\end{array}$$

in which *t*_*i*_ is the concentration (in SQU/ml) for which *D*_*i*_ is measured;

$${{t0}} = 100,\,{{t}}1 = 330,\,{{t}}2 = 1000,\,{{t}}3 = 3300,\,{{t}}4 = 10,000,\,{{\rm and}}\,{{t}}5 = 33,000;$$

*D*_*i*_ is the average wheal diameter obtained at concentration *t*_*i*_.

The negative control was not subtracted from this value.

### Safety

Safety assessments included rates of TEAEs or serious AEs (SAEs) through day 85 as reported by investigators, along with vital signs and laboratory tests. Adverse events were described at the Medical Dictionary for Regulatory Activities (MedDRA; version 17.0) to lowest level terms.

### Clinical statistical analysis

A sample size of approximately 70 patients with cat allergy was needed to provide at least 90% power to detect the expected mean differences in primary endpoint between the two treatment groups, with an assumption of a TNSS AUC mean value of 5 for the placebo group and 3 for the treatment group during the course of 0–1-h post-challenge. The assumed standard deviation of 2.43 is consistent with the effect of topical nasal corticosteroids^69,70^. Primary efficacy analyses were conducted in the FAS which includes all randomized patients who received any study drug on day 1 and had TNSS evaluation results on day 8 (*n* = 36 and 34, for placebo and REGN1908–1909 groups, respectively). Efficacy endpoints were analyzed using analysis-of-covariance (ANCOVA) model with treatment group as a factor and baseline value as a covariate. The results of the ANCOVA model included the summary of least-squares (LS) mean for each treatment group with corresponding standard error (SE), the LS mean difference between treatment group with corresponding SE and 95% confidence interval (CI), and the *p*-value corresponding to the between-treatment-group difference. Sensitivity analyses were performed for all efficacy analyses by excluding four patients from a study site terminated early due to non-compliance, as determined by the investigator/sponsor, and for the primary efficacy endpoint by imputing three missing TNSS AUC(0–1 h) values at day 8 with baseline values. Secondary and exploratory efficacy endpoints were analyzed using the same ANCOVA model as the primary analysis; no control for multiplicity was performed for secondary and exploratory endpoints, therefore *p* values are considered nominal. The safety analysis set included all randomized patients who received any study drug on day 1, based on the treatment received.

### Data availability

The authors declare that the data supporting the findings of this study are available within the article and its supplementary information files, or are available upon reasonable requests to the authors.

## Electronic supplementary material

Supplementary Information(PDF 1549 kb)

## References

[1] Hansen I, Klimek L, Mosges R, Hormann K (2004). Mediators of inflammation in the early and the late phase of allergic rhinitis. Curr. Opin. Allergy Clin. Immunol..

[2] Durham SR (2012). SQ-standardized sublingual grass immunotherapy: confirmation of disease modification 2 years after 3 years of treatment in a randomized trial. J. Allergy Clin. Immunol..

[3] Durham SR (1999). Long-term clinical efficacy of grass-pollen immunotherapy. N. Engl. J. Med..

[4] Verhoef A, Alexander C, Kay AB, Larche M (2005). T cell epitope immunotherapy induces a CD4+T cell population with regulatory activity. PLoS. Med..

[5] Akdis CA (1996). Epitope-specific T cell tolerance to phospholipase A2 in bee venom immunotherapy and recovery by IL-2 and IL-15 in vitro. J. Clin. Invest..

[6] Ryan JF (2016). Successful immunotherapy induces previously unidentified allergen-specific CD4+T-cell subsets. Proc. Natl Acad. Sci. USA.

[7] Ebner C (1997). Immunological changes during specific immunotherapy of grass pollen allergy: reduced lymphoproliferative responses to allergen and shift from TH2 to TH1 in T-cell clones specific for Phl p 1, a major grass pollen allergen. Clin. Exp. Allergy.

[8] Circassia. Circassia announces top-line results from cat allergy phase III study. http://www.circassia.com/media/press-releases/circassia-announces-top-line-results-from-cat-allergy-phase-III-study. Accessed: 30 June 2016 (2016).

[9] Wachholz PA, Durham SR (2004). Mechanisms of immunotherapy: IgG revisited. Curr. Opin. Allergy Clin. Immunol..

[10] Nanda A (2004). Dose dependence and time course of the immunologic response to administration of standardized cat allergen extract. J. Allergy Clin. Immunol..

[11] Jones SM (2009). Clinical efficacy and immune regulation with peanut oral immunotherapy. J. Allergy Clin. Immunol..

[12] Francis JN (2008). Grass pollen immunotherapy: IL-10 induction and suppression of late responses precedes IgG4 inhibitory antibody activity. J. Allergy Clin. Immunol..

[13] Jarolim E (1990). A long-term follow-up study of hyposensitization with immunoblotting. J. Allergy Clin. Immunol..

[14] Shamji MH (2012). Functional rather than immunoreactive levels of IgG4 correlate closely with clinical response to grass pollen immunotherapy. Allergy.

[15] James LK (2011). Long-term tolerance after allergen immunotherapy is accompanied by selective persistence of blocking antibodies. J. Allergy Clin. Immunol..

[16] Kleine-Tebbe J (1993). Role of the major allergen (Fel d I) in patients sensitized to cat allergens. Int. Arch. Allergy Immunol..

[17] Macdonald LE (2014). Precise and in situ genetic humanization of 6 Mb of mouse immunoglobulin genes. Proc. Natl. Acad. Sci. USA.

[18] Murphy AJ (2014). Mice with megabase humanization of their immunoglobulin genes generate antibodies as efficiently as normal mice. Proc. Natl Acad. Sci. USA.

[19] Yang X, Ambrogelly A (2014). Enlarging the repertoire of therapeutic monoclonal antibodies platforms: domesticating half molecule exchange to produce stable IgG4 and IgG1 bispecific antibodies. Curr. Opin. Biotechnol..

[20] Liu Y, Zhu M, Nishida K, Hirano T, Zhang W (2007). An essential role for RasGRP1 in mast cell function and IgE-mediated allergic response. J. Exp. Med..

[21] Ocmant A (2007). Flow cytometry for basophil activation markers: the measurement of CD203c up-regulation is as reliable as CD63 expression in the diagnosis of cat allergy. J. Immunol. Methods.

[22] Gilfillan AM, Tkaczyk C (2006). Integrated signalling pathways for mast-cell activation. Nat. Rev. Immunol..

[23] Scadding GW (2015). Local and systemic effects of cat allergen nasal provocation. Clin. Exp. Allergy.

[24] Baroody FM, Shenaq D, DeTineo M, Wang J, Naclerio RM (2009). Fluticasone furoate nasal spray reduces the nasal-ocular reflex: a mechanism for the efficacy of topical steroids in controlling allergic eye symptoms. J. Allergy Clin. Immunol..

[25] Corren J (1999). Onset and duration of action of levocabastine nasal spray in atopic patients under nasal challenge conditions. J. Allergy Clin. Immunol..

[26] Eckman JA (2010). Effects of omalizumab on basophil and mast cell responses using an intranasal cat allergen challenge. J. Allergy Clin. Immunol..

[27] Ewbank PA (2003). A double-blind, placebo-controlled immunotherapy dose–response study with standardized cat extract. J. Allergy Clin. Immunol..

[28] Pawankar, R., Holgate S. T., Canonica, G. W. & Lockey, R. (eds) *White Book on Allergy* (World Allergy Organization, Milwaukee, WI, 2011).

[29] Noon, L. Prophylactic inoculation against hay fever. *Lancet* **177**, 1572–1573 (1911).

[30] Valenta R, Campana R, Focke-Tejkl M, Niederberger V (2016). Vaccine development for allergen-specific immunotherapy based on recombinant allergens and synthetic allergen peptides: Lessons from the past and novel mechanisms of action for the future. J. Allergy Clin. Immunol..

[31] Larche M (2011). T cell epitope-based allergy vaccines. Curr. Top. Microbiol. Immunol..

[32] O’Hehir RE, Prickett SR, Rolland JM (2016). T cell epitope peptide therapy for allergic diseases. Curr. Allergy Asthma Rep..

[33] Worm M (2011). Development and preliminary clinical evaluation of a peptide immunotherapy vaccine for cat allergy. J. Allergy Clin. Immunol..

[34] Couroux P, Patel D, Armstrong K, Larche M, Hafner RP (2015). Fel d 1-derived synthetic peptide immuno-regulatory epitopes show a long-term treatment effect in cat allergic subjects. Clin. Exp. Allergy.

[35] Patel D (2013). Fel d 1-derived peptide antigen desensitization shows a persistent treatment effect 1 year after the start of dosing: a randomized, placebo-controlled study. J. Allergy Clin. Immunol..

[36] Circassia. Circassia aunnounces top-line results from house dust mite allergy field study. http://www.circassia.com/media/press-releases/circassia-announces-top-line-results-from-house-dust-mite-allergy-field-study/ Accessed: 19 April 2017 (2017).

[37] Spertini F (2016). Efficacy of 2 months of allergen-specific immunotherapy with Bet v 1-derived contiguous overlapping peptides in patients with allergic rhinoconjunctivitis: results of a phase IIb study. J. Allergy Clin. Immunol..

[38] Focke-Tejkl M (2015). Development and characterization of a recombinant, hypoallergenic, peptide-based vaccine for grass pollen allergy. J. Allergy Clin. Immunol..

[39] Zieglmayer P (2016). Mechanisms, safety and efficacy of a B cell epitope-based vaccine for immunotherapy of grass pollen allergy. EBioMedicine.

[40] Curin M (2014). Hypoallergenic derivatives of Fel d 1 obtained by rational reassembly for allergy vaccination and tolerance induction. Clin. Exp. Allergy.

[41] Flicker S, Linhart B, Wild C, Wiedermann U, Valenta R (2013). Passive immunization with allergen-specific IgG antibodies for treatment and prevention of allergy. Immunobiology.

[42] Flicker S, Valenta R (2003). Renaissance of the blocking antibody concept in type I allergy. Int. Arch. Allergy Immunol..

[43] Scadding GW (2017). Effect of 2 years of treatment with sublingual grass pollen immunotherapy on nasal response to allergen challenge at 3 years among patients with moderate to severe seasonal allergic rhinitis: The GRASS randomized clinical trial. JAMA.

[44] Durham SR (2016). Treatment effect of sublingual immunotherapy tablets and pharmacotherapies for seasonal and perennial allergic rhinitis: pooled analyses. J. Allergy Clin. Immunol..

[45] Du Toit G (2016). Effect of avoidance on peanut allergy after early peanut consumption. N. Engl. J. Med..

[46] Casale TB (2001). Effect of omalizumab on symptoms of seasonal allergic rhinitis: a randomized controlled trial. JAMA.

[47] Ong YE (2005). Anti-IgE (omalizumab) inhibits late-phase reactions and inflammatory cells after repeat skin allergen challenge. J. Allergy Clin. Immunol..

[48] Corren J (2008). Allergen skin tests and free IgE levels during reduction and cessation of omalizumab therapy. J. Allergy Clin. Immunol..

[49] Wenzel S (2016). Dupilumab efficacy and safety in adults with uncontrolled persistent asthma despite use of medium-to-high-dose inhaled corticosteroids plus a long-acting beta2 agonist: a randomised double-blind placebo-controlled pivotal phase 2b dose-ranging trial. Lancet.

[50] Beck LA (2014). Dupilumab treatment in adults with moderate-to-severe atopic dermatitis. N. Engl. J. Med..

[51] Hamilton JD (2014). Dupilumab improves the molecular signature in skin of patients with moderate-to-severe atopic dermatitis. J. Allergy Clin. Immunol..

[52] Simpson EL (2016). Two phase 3 trials of dupilumab versus placebo in atopic dermatitis. N. Engl. J. Med..

[53] Bachert C (2016). Effect of subcutaneous dupilumab on nasal polyp burden in patients with chronic sinusitis and nasal polyposis: a randomized clinical trial. JAMA.

[54] Paterniti M (2011). Cat allergen-induced blood basophil reactivity in vitro predicts acute human nasal allergen challenge responses in vivo. Clin. Exp. Allergy.

[55] Shamji MH (2006). The IgE-facilitated allergen binding (FAB) assay: validation of a novel flow-cytometric based method for the detection of inhibitory antibody responses. J. Immunol. Methods.

[56] Flicker S, Gadermaier E, Madritsch C, Valenta R (2011). Passive immunization with allergen-specific antibodies. Curr. Top. Microbiol. Immunol..

[57] Zhu D (2005). A chimeric human-cat fusion protein blocks cat-induced allergy. Nat. Med..

[58] Uermosi C (2014). IgG-mediated down-regulation of IgE bound to mast cells: a potential novel mechanism of allergen-specific desensitization. Allergy.

[59] Wickman M (2005). When allergies complicate allergies. Allergy.

[60] Nopp A, Johansson SG, Lundberg M, Oman H (2006). Simultaneous exposure of several allergens has an additive effect on multisensitized basophils. Allergy.

[61] Niespodziana K (2011). A hypoallergenic cat vaccine based on Fel d 1-derived peptides fused to hepatitis B PreS. J. Allergy Clin. Immunol..

[62] Yasueda H, Yui Y, Shimizu T, Shida T (1983). Isolation and partial characterization of the major allergen from Japanese cedar (Cryptomeria japonica) pollen. J. Allergy Clin. Immunol..

[63] Gobl, C. et al. Flexible IgE epitope containing domains of Phl p 5 cause high allergenic activity. *J Allergy Clin Immunol* **140**, 1187–1191 (2017). 10.1016/j.jaci.2017.05.005 PMC5632575 28532654

[64] Kazemi-Shirazi L (2002). Recombinant marker allergens: diagnostic gatekeepers for the treatment of allergy. Int. Arch. Allergy Immunol..

[65] Kaiser L (2003). The crystal structure of the major cat allergen Fel d 1, a member of the secretoglobin family. J. Biol. Chem..

[66] McCoy AJ (2007). Phaser crystallographic software. J. Appl. Crystallogr..

[67] Ye JD (2008). Synthetic antibodies for specific recognition and crystallization of structured RNA. Proc. Natl Acad. Sci. USA.

[68] Emsley P, Lohkamp B, Scott WG, Cowtan K (2010). Features and development of Coot. Acta Crystallogr. D: Biol. Crystallogr..

[69] Wu W, Walters RD, Nadeau GA, Botnick W, Broughton N (2013). An integrated analysis of the efficacy of fluticasone furoate nasal spray versus placebo on the nasal symptoms of perennial allergic rhinitis. Allergy Asthma Proc..

[70] Nicholson GC (2011). The effects of an anti-IL-13 mAb on cytokine levels and nasal symptoms following nasal allergen challenge. J. Allergy Clin. Immunol..

