# Supplementary material for: Treating cat allergy with monoclonal IgG antibodies that bind allergen and prevent IgE engagement
Source: Nat Commun. 2018 Apr 12;9:1421. doi: 10.1038/s41467-018-03636-8 (PMC5897525; doi:10.1038/s41467-018-03636-8)
Supplement: Supplementary file 1 — Supplementary Information(PDF 1549 kb) [file 41467_2018_3636_MOESM1_ESM.pdf]

Supplementary Information

Treating cat allergy with monoclonal IgG antibodies that bind allergen and prevent IgE engagement

Orengo, et al.

<u>Supplementary Materials</u>

Supplementary Table 1: Summary of Binding Parameters for the Interaction of Cat-SIT-IgG with nFel d 1

Supplementary Table 2: Demographics and Baseline Disease Characteristics

Supplementary Table 3: Sensitivity Analyses for Primary Efficacy Endpoint

Supplementary Table 4: TNSS AUC(1-8hr) Absolute Change from Baseline

Supplementary Table 5: TNSS AUC(0-1hr) Component Analysis on Study Day 8

Supplementary Table 6: Peak Nasal Inspiratory Flow Absolute Change from Baseline

Supplementary Table 7: Summary of Safety Results

Supplementary Table 8: TNSS AUC(0-1hr) Absolute Change from Baseline Days 29, 57, 85

Supplementary Table 9: X-ray Crystallography Data Collection and Refinement Statistics

Supplementary Figure 1: Binding Characteristics of REGN1908 and REGN1909

Supplementary Figure 2: Dose-response curves for basophils from individual cat-allergic donors

Supplementary Figure 3: Analysis of Cat Hair Extract Components

Supplementary Figure 4: Cat-SIT-IgG PCA Mouse Model Supporting Data

Supplementary Figure 5: Effector Cell Gating Strategies

Supplementary Figure 6: Schematic of Nasal Allergen Challenge Procedure

Supplementary Figure 7: Subject Disposition

Supplementary Figure 8: Absolute Wheal Diameter for the Titrated Cat Hair Extract Skin Prick Test

SUPPLEMENTARY TABLES

Supplementary Table 1. Summary of binding parameters for the interaction of Cat-SIT-IgG with rFel d 1.mmh

Sample Injected (Donor ID)	k_a ($M^{-1}s^{-1}$)	k_d (s^{-1})	K_D (M)	$t_{½}$ (min)
2-02	2.53×10^{5}	9.87×10^{-4}	3.90×10^{-9}	12
2-03 (5)*	2.14×10^{5}	5.77×10^{-4}	2.69×10^{-9}	20
2-04	1.14×10^{5}	$5.00 \times 10^{-5\#}$	4.40×10^{-10}	$231^{\#}$
2-05 (7)*	1.80×10^{5}	5.59×10^{-4}	3.11×10^{-9}	21
2-06	3.81×10^{5}	1.97×10^{-3}	5.17×10^{-9}	6
2-07 (10)*	2.42×10^{5}	9.48×10^{-4}	3.92×10^{-9}	12
2-08	1.48×10^{5}	2.39×10^{-4}	1.61×10^{-9}	48
2-09	1.41×10^{5}	1.38×10^{-3}	9.81×10^{-9}	8
2-10	1.40×10^{5}	$5.00 \times 10^{-5\#}$	3.60×10^{-10}	$231^{\#}$
Control Ab	NB	NB	NB	NB

Patient IDs with * denote 3 patients who donated blood at two visits. Samples from the first visit were used in the passive cutaneous anaphylaxis (PCA) Model, Figure 4. Binding parameters presented here for these 3 patients are from sample obtained from their second visit. The number in parentheses represents donor ID from the first visit.

Cat-SIT-IgG: IgG isolated from sera of patients who underwent physician-defined successful cat specific immunotherapy as described in Methods; rFel d 1.mmh: Recombinant Fel d 1 with a myc-myc-hexahistidine tag; k_a: association rate constant; k_d: dissociation rate constant; K_D: equilibrium dissociation constant; NB: no binding observed under the assay conditions; $t_{½}$: dissociative half-life. # indicates that no dissociation of Cat-SIT-IgG from rFeld1.mmh surface was observed and based on the limitation of the experimental condition k_d value was fixed at 5.00×10^{-5} s^{-1} during analysis.

Supplementary Table 2. Demographics and Baseline Disease Characteristics

	**Placebo (n=37)**	**REGN1908-1909 600mg (n=36)**
**Demographics**		
Age, mean ± SD, y	27.6 ± 9.49	28.1 ± 9.80
Male/Female, n	18/19	18/18
Race		
White, n(%)	33 (89.2)	29 (80.6)
Asian, n (%)	3 (8.1)	4 (11.1)
Other, n(%)	1 (2.7)	3 (8.3)
BMI, mean ± SD, kg/m^2	23.90 ± 2.67	24.24 ± 3.42
**Disease history**		
Anti-Fel d 1 IgE titer, median (range), kU/L	3.70 (0.2 : 76.8)	1.99 (0.3 : 55.8)
Anti-Cat dander IgE titer, median (range), kU/L	3.93 (0.4 : 56.2)	1.76 (0.3 : 81.1)
Wheal cat hair extract SPT, mean ± SD, mm	7.63 (2.841)	7.25 (2.746)
Number of other allergens positive by SPT, n (type)		
None	5	6
1	5 (4A, 1P)	6 (5A, 1P)
2	14 (2A, 7P, 5AP)	10 (1A, 7P, 2AP)
≥3	13 (13AP)	14 (14AP)
Number of other allergens positive by IgE, n (type)		
None	1	2
1	5 (5P)	5 (5P)
2	10 (1A, 7P, 2AP)	11 (2A, 7P, 2AP)
≥3	21 (1P, 20 AP)	18 (3P, 15AP)

IgE titers presented are baseline values collected at day 1, i.e. the day of dosing.
For positivity to allergens other than cat, a patient was considered IgE-positive if titers were >0.35kU/L, and considered SPT-positive if wheal diameter was >3mm greater than the negative control. Fel d 2 was included in the panel (2 subjects were positive to Fel d 2 in each group). The allergen sets used for SPT and IgE testing were not fully overlapping, and included prominent regional allergens based on study site location.

SD: Standard Deviation; BMI: body mass index; SPT: skin prick test
Allergen Types: A: Aeroallergen; P: Perennial allergen; AP: allergic to both types

Supplementary Table 3. Sensitivity Analyses for Primary Efficacy Endpoint

Absolute change from baseline to day 8 NAC in TNSS AUC(0-1h)

**Full Analysis Set Excluding Site 528101**			
	**Placebo (n=33)**	**REGN1908-1909 (n=33)**	***P* value**
Baseline Mean (SD)	5.758 (1.194)	5.662 (1.432)	
Day 8 Mean (SD)	4.237 (1.919)	2.404 (2.064)	
Change from baseline LS mean (SE)	-1.481 (0.347)	-3.297 (0.347)	
LS mean difference vs placebo (SE)		-1.816 (0.491)	0.0005
95%CI		(-2.798, -0.834)	

**All randomized subjects, Baseline Observation Carried Forward**			
	**Placebo (n=37)**	**REGN1908-1909 (n=36)**	***P* value**
Baseline Mean (SD)	5.610 (1.227)	5.641 (1.423)	
Day 8 Mean (SD)	4.146 (1.863)	2.512 (2.035)	
Change from baseline LS mean (SE)	-1.477 (0.321)	-3.117 (0.325)	
LS mean difference vs placebo (SE)		-1.640 (0.457)	
95%CI		(-2.551, -0.729)	0.0006

Analyses are based on ANCOVA model with treatment group as a factor and baseline as a covariate.

NAC: Nasal allergen challenge; TNSS: Total nasal symptom score; AUC(0-1h): Area under the curve (hour 0 to hour 1); SD: Standard deviation; LS Mean: Least squares mean; SE: Standard error of the LS Mean; CI: Confidence interval.

Supplementary Table 4. TNSS AUC(1-8h) Absolute Change from Baseline

	Placebo (n=36)	REGN1908-1909 (n=34)	*P* value
**Absolute change from baseline to day 8 in TNSS AUC (1-8)**			
Pre-treatment Mean (SD)	1.667 (1.174)	1.202 (1.301)	
Day 8 Mean (SD)	1.486 (1.574)	0.542 (0.773)	
Change from baseline LS mean (SE)	-0.058 (0.189)	-0.789 (0.195)	
LS mean difference vs placebo (SE)		-0.731 (0.274)	
95%CI		(-1.278, -0.185)	0.0094
**Absolute change from baseline to day 29, 57, 85 in TNSS AUC(1-8)**			
Change from baseline to day 29 LS mean (SE)	-0.072 (0.197)	-0.852 (0.197)	
LS mean difference vs placebo (SE)		-0.780 (0.282)	
95%CI		(-1.343,-0.216)	0.0074
Change from baseline to day 57 LS mean (SE)	-0.471 (0.211)	-0.289 (0.214)	
LS mean difference vs placebo (SE)		-0.182 (0.304)	
95%CI		(-0.425, 0.789)	0.5503
Change from baseline to day 85 LS mean (SE)	-0.268 (0.190)	-0.652 (0.190)	
LS mean difference vs placebo (SE)		-0.383 (0.272)	
95%CI		(-0.926, 0.160)	0.1636

Analyses are based on ANCOVA model with treatment group as a factor and baseline as a covariate. For secondary efficacy endpoints no control for multiplicity was performed, therefore p values are considered nominal.

TNSS: Total nasal symptom score; AUC(1-8): Area under the curve (hour 1 to hour 8); SD: Standard deviation; LS Mean: Least squares mean; SE: Standard error of the LS Mean; CI: Confidence interval.

**Supplementary Table 5.** TNSS AUC(0-1hr) Component Analysis on Study Day 8				
**TNSS Component Test**	**Placebo (n=36) Mean Change from Baseline in AUC (0-1h)**	**REGN1908-1909 (n=34) Mean Change from Baseline in AUC (0-1h)**	**Treatment Difference in Change from Baseline AUC(0-1h)**	**Percent of Total Score**
Nasal Congestion	-0.44	-1.05	-0.61	34%
Nasal Itching	-0.58	-0.84	-0.27	15%
Rhinorrhea	-0.36	-1.07	-0.71	39%
Sneezing	-0.14	-0.36	-0.22	12%
Total Score	-1.51	-3.32	-1.80	
AUC(0-1h): Area under the curve (hour 0 to hour 1).				

**Supplementary Table 6.** Peak Nasal Inspiratory Flow AUC(0-1h) Absolute Change from Baseline			
	**Placebo (n=36)**	**REGN1908-1909 (n=34)**	***P* value**
**Absolute change from baseline to day 8 in PNIF AUC (0-1)**			
Pre-treatment Mean (SD)	88.900 (71.808)	67.828 (55.978)	
Day 8 Mean (SD)	89.132 (50.217)	96.164 (40.439)	
Change from baseline LS mean (SE)	7.168 (6.882)	20.991 (7.085)	
LS mean difference vs placebo (SE)		13.823 (9.944)	
95%CI		(-6.025, 33.670)	0.1691
**Absolute change from baseline to day 29, 57, 85 in PNIF AUC (0-1)**			
Change from baseline to day 29 LS mean (SE)	2.077 (6.554)	18.660 (6.554)	
LS mean difference vs placebo (SE)		16.583 (9.301)	
95%CI		(-1.992, 35.159)	0.0793
Change from baseline to day 57 LS mean (SE)	12.428 (6.266)	15.702 (6.364)	
LS mean difference vs placebo (SE)		3.274 (8.954)	
95%CI		(-14.625, 21.173	0.7159
Change from baseline to day 85 LS mean (SE)	6.115 (6.063)	21.928 (6.063)	
LS mean difference vs placebo (SE)		15.813 (8.602)	
95%CI		(-1.378, 33.003)	0.0708

Analyses are based on ANCOVA model with treatment group as a factor and baseline as a covariate. For secondary efficacy endpoints no control for multiplicity was performed, therefore p values are considered nominal.

PNIF: Peak nasal inspiratory flow; AUC(0-1h): Area under the curve (hour 0 to hour 1); SD: Standard deviation; LS Mean: Least squares mean; SE: Standard error of the LS Mean; CI: Confidence interval.

Supplementary Table 7. Summary of Safety Results

n (%)	Placebo (n=37)	REGN1908-1909 600mg (n=36)
**Overall results**		
Subjects with any TEAE	23 (62.2)	23 (63.9)
Subjects with any SAE	1 (2.7)[a]	1 (2.8)[b]
Subjects with any TEAE that led to treatment discontinuation	0	0
Subjects with any TEAE that led to death	0	0
**Treatment-Emergent Adverse Events (>3% in any group)**		
Infections	12 (32.4)	14 (38.9)
Upper respiratory tract infection	4 (10.8)	5 (13.9)
Viral upper respiratory tract infection	2 (5.4)	2 (5.6)
Rhinitis	1 (2.7)	3 (8.3)
Gastroenteritis	0	2 (5.6)
Nasopharyngitis	3 (8.1)	1 (2.8)
Headache	5 (13.5)	6 (16.7)
Dizziness	1 (2.7)	2 (5.6)
Nausea	1 (2.7)	3 (8.3)
Abdominal pain	2 (5.4)	2 (5.6)
Vomiting	1 (2.7)	2 (5.6)
Cough	1 (2.7)	3 (8.3)
Nasal congestion	2 (5.4)	0
Eczema	2 (5.4)	0

TEAE, treatment-emergent adverse event; SAE, serious adverse event.
[a]One subject in the placebo group was categorized as appendicitis, non-treatment related per investigator assessment; [b]One subject in the REGN19008-1909 group was categorized as pyelonephritis, non-treatment related per investigator assessment.

Supplementary Table 8. TNSS AUC(0-1h) Absolute Change from Baseline for Days 29, 57, 85

	**Placebo (n=36)**	**REGN1908-1909 (n=34)**	***P* value**
Change from baseline to day 29 LS mean (SE)	-1.586 (0.3392)	-3.663 (0.3392)	
LS mean difference vs placebo (SE)		-2.077 (0.4798)	
95%CI		(-3.035,-1.118)	<0.0001
Change from baseline to day 57 LS mean (SE)	-2.098 (0.3625)	-3.045 (0.3681)	
LS mean difference vs placebo (SE)		-0.947 (0.5167)	
95%CI		(-1.980, 0.086)	0.0718
Change from baseline to day 85 LS mean (SE)	-1.660 (0.3595)	-3.109 (0.3595)	
LS mean difference vs placebo (SE)		-1.448 (0.5085)	
95%CI		(-2.465,-0.432)	0.0059

Analyses are based on ANCOVA model with treatment group as a factor and baseline as a covariate. For secondary efficacy endpoints no control for multiplicity was performed, therefore p values are considered nominal.

TNSS: Total nasal symptom score; AUC(0-1h): Area under the curve (hour 0 to hour 1); SD: Standard deviation; LS Mean: Least squares mean; SE: Standard error of the LS Mean; CI: Confidence interval.

**Supplementary Table 9.** X-ray Crystallography Data Collection and Refinement Statistics		
**Data Collection Statistics**		
Space group	$P2_12_12_1$	
Unit cell (Å)	74.98 131.48 148.36	
Data collection location	Beamline 5.0.2, Advanced Light Source, LBNL	
Resolution (Å)	100 - 2.9	2.95 - 2.90
Unique reflections	33144	1596
R_{pim}	0.055	0.446
$CC_{1/2}$	---	0.75
Completeness (%)	99.9	99.8
Average redundancy	7.2	6.9
I/ ∑ (I)	14.9	1.6
**Refinement Statistics**		
Resolution (Å)	100 - 2.9	
Reflections used (free)	30883 (1643)	
R factor	0.225	
Rfree	0.259	
RMSD		
bonds (Å)	0.007	
angles (°)	1.17	
Ramachandran statistics		
Most favored (%)	97.7	
Additional allowed (%)	1.6	
Outliers (%)	0.7	
Rotameric outliers (%)	3.9	
Asymmetric unit contents	2 complexes of Fab + single-chain Fel d1	
PDB code	5VYF	

SUPPLEMENTARY FIGURES

a

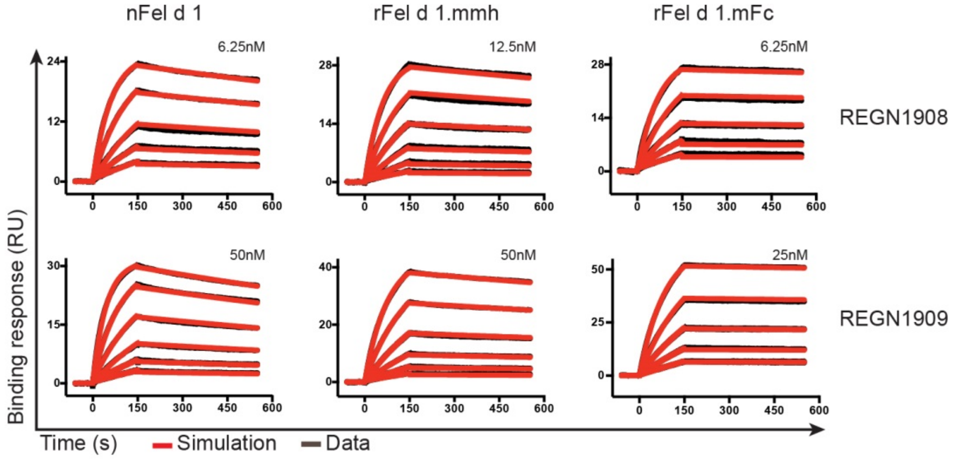

b

Test Ligand	Binding Parameters for REGN1908			
	k_a ($M^{-1}s^{-1}$)	k_d (s^{-1})	K_D (M)	$t_{1/2}$ (min)
rFel d 1.mmh (monomeric)	1.34×10^{6}	2.51×10^{-4}	1.87×10^{-10}	46
rFel d 1.mFc (dimeric)	2.30×10^{6}	7.25×10^{-5}	3.16×10^{-11}	159
nFel d 1	2.58×10^{6}	3.59×10^{-4}	1.39×10^{-10}	32
**Test Ligand**	**Binding Parameters for REGN1909**			
	k_a ($M^{-1}s^{-1}$)	k_d (s^{-1})	K_D (M)	$t_{1/2}$ (min)
rFel d 1.mmh (monomeric)	2.57×10^{5}	2.35×10^{-4}	9.12×10^{-10}	49
rFel d 1.mFc (dimeric)	4.66×10^{5}	4.55×10^{-5}	9.78×10^{-11}	254
nFel d 1	4.31×10^{5}	4.48×10^{-4}	1.04×10^{-9}	26

c

Step 1: Amine-coupled anti-Fel d 1 mAb1	Step 2: Capture nFel d 1	Step 3: Monitor binding of 25 µg/mL of mAb2 to the mAb1:Fel d 1 surface		
anti-Fel d 1 mAb1	Antigen Capture 200nM nFel d 1 (RU)	Bound REGN1908 (RU)	Bound REGN1909 (RU)	Non-binding Control mAb (RU)
**REGN1908**	125 ± 4	4	**483**	-5
**REGN1909**	541 ± 5	**1831**	-63	-64

Supplementary Figure 1: Binding characteristics of REGN1908 and REGN1909. a REGN1908 or REGN1909 were captured using a polyclonal goat anti-human Fcγ antibody-immobilized sensor surface followed by injection of different concentrations of nFel d 1, rFel d 1.mmh, or rFel d 1.mFc were two-fold serially diluted in $HBSP^{++}$ buffer. Binding studies were performed at 25°C and the real-time binding sensorgrams of nFel d 1, rFel d 1.mmh, or rFel d 1.mFc are shown in black, while global fits obtained using 1:1 binding model with mass transport limitation are represented by red lines. The highest Fel d 1 concentration used to fit the data is provided and results of the kinetic analysis are tabulated in (b). **b** Binding kinetics table for the interaction of REGN1908 and REGN1909 with nFel d 1, rFel d 1.mmh, or rFel d 1.mFc. **c** Binding response measured for sequential binding of REGN1908 and REGN1909 (presented in Figure 2A) supporting their simultaneous binding to nFel d 1. nFel d 1: Natural Fel d 1; rFel d 1.mmh: Recombinant Fel d 1 with a myc-myc-hexahistidine tag; rFel d 1.mFc: Recombinant Fel d 1 with a mouse IgG2a Fc (mFc) tag; k_a: association rate constant; k_d: dissociation rate constant; K_D: equilibrium dissociation constant; $t_{1/2}$: dissociative half-life.

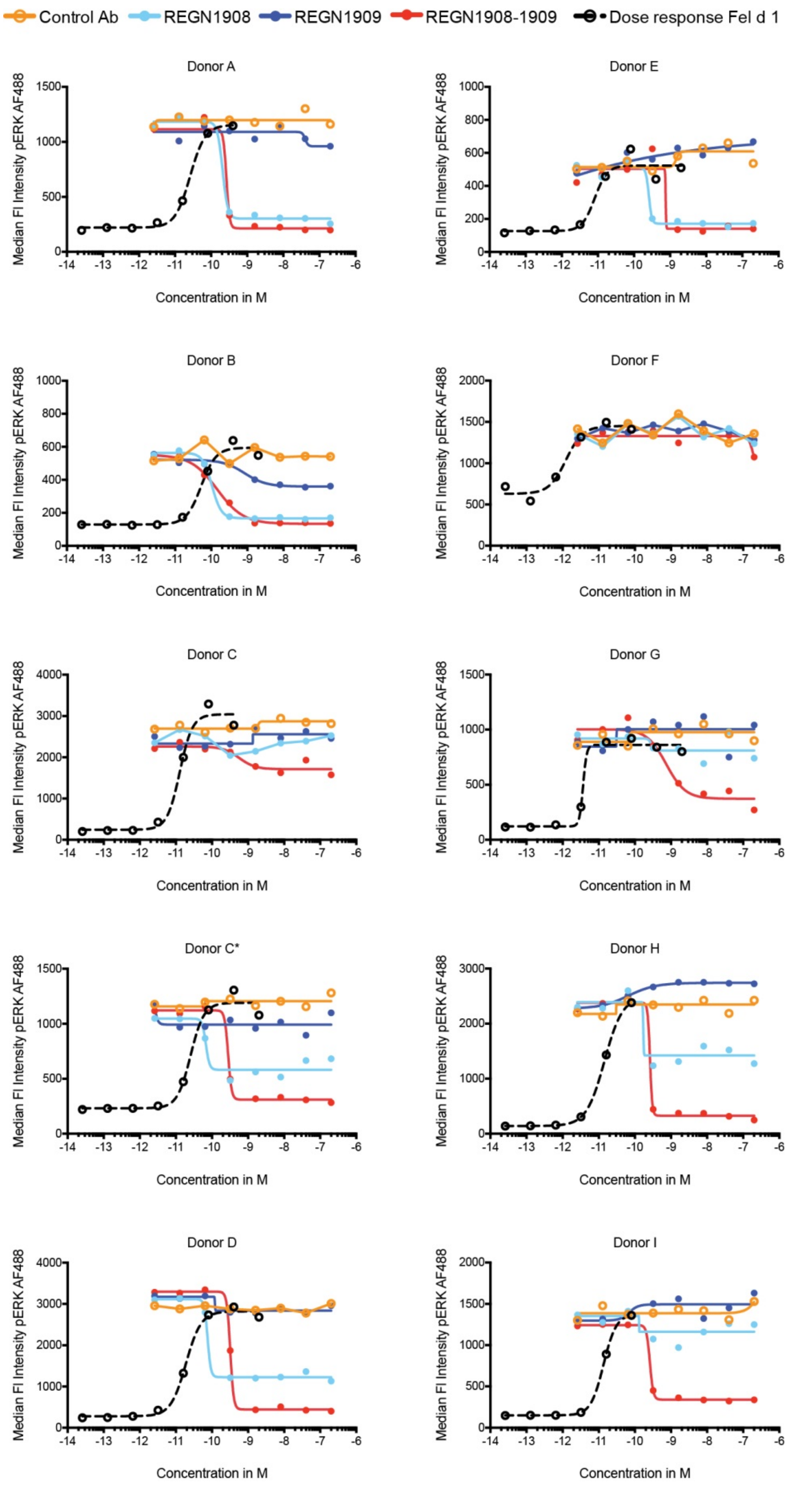

Supplementary Figure 2. Dose-response curves for basophils from individual cat-allergic donors. The ability of REGN1908, REGN1909 or REGN1908-1909 to block natural Fel d 1 (nFel d 1)-induced activation of basophils from cat allergic donors was measured as pErk response to 200pM nFel d 1 stimulation. Basophils were isolated from 10 cat allergic donors using flow cytometry analysis gating on basophils (CD123^{+} HLA-DR^{-}) [Supplemental Fig. 5]. Antibodies were added in a single-point dose response ranging from 2.56fM to 200nM. C and C* represent independent blood donations from the same donor 2 months apart.

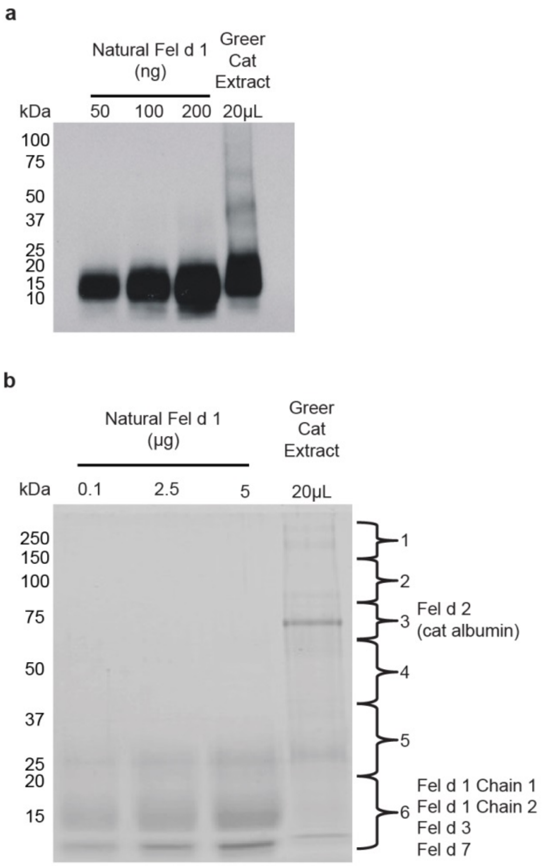

Supplementary Figure 3: Analysis of cat hair extract components. a Immunoblot of natural Fel d 1 and Greer Cat Hair extract run on a tris-glycine 4-20% reducing SDS-PAGE gel followed by visualization with anti-Fel d 1 and anti-human horseradish peroxidase (HRP). **b** Greer cat hair extract was subjected to SDS-PAGE under reducing conditions followed by nanoLC-MS/MS analysis of peptides derived from in-gel trypsin digestion of six gel sections covering the entire gel lane. The MS/MS spectra were searched against non-redundant SwissProt protein database by the Mascot search engine. A total of 69 proteins were identified with false discovery rate less than 1%. Fel d 1 Chain 1 and Chain 2 were identified with five and three unique peptide sequences, respectively in gel section 6 (data not shown) corresponding to proteins migrating below a 20 kDa molecular weight marker, confirming the presence of Fel d 1 in the sample.

a

Patient #	SIT status (duration in months)	Total concentrated IgG (mg/mL)	Concentrated Fel d 1 specific IgG (mg/mL)	% of purified IgG that is Fel d 1 specific
003	No	107.3	0.007	0.007
004	No	112.3	0.007	0.006
002	Yes (27)	61.7	0.078	0.126
005	Yes (13)	131.1	0.059	0.045
007	Yes (20)	146.4	0.120	0.082
008	Yes (20)	67.1	0.015	0.022
010	Yes (35)	161.9	0.463	0.286

b

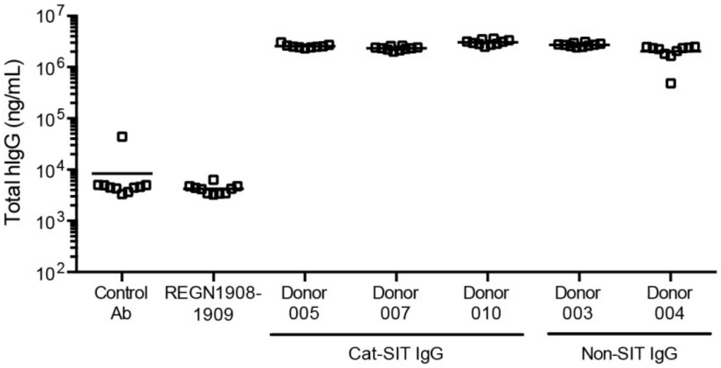

Supplementary Figure 4: Cat-SIT-IgG Passive Cutaneous Anaphylaxis (PCA) mouse model supporting data. a Characteristics of patients whose sera was used in the PCA model, Figure 4. Antibody concentrations were determined by ELISA. **b** Circulating human antibody levels in the serum of mice challenged in the PCA model (Figure 4b) collected at sacrifice (day 5). Cat-SIT-IgG: IgG isolated from sera of patients who underwent physician-defined successful cat specific immunotherapy (SIT) as described in Methods.

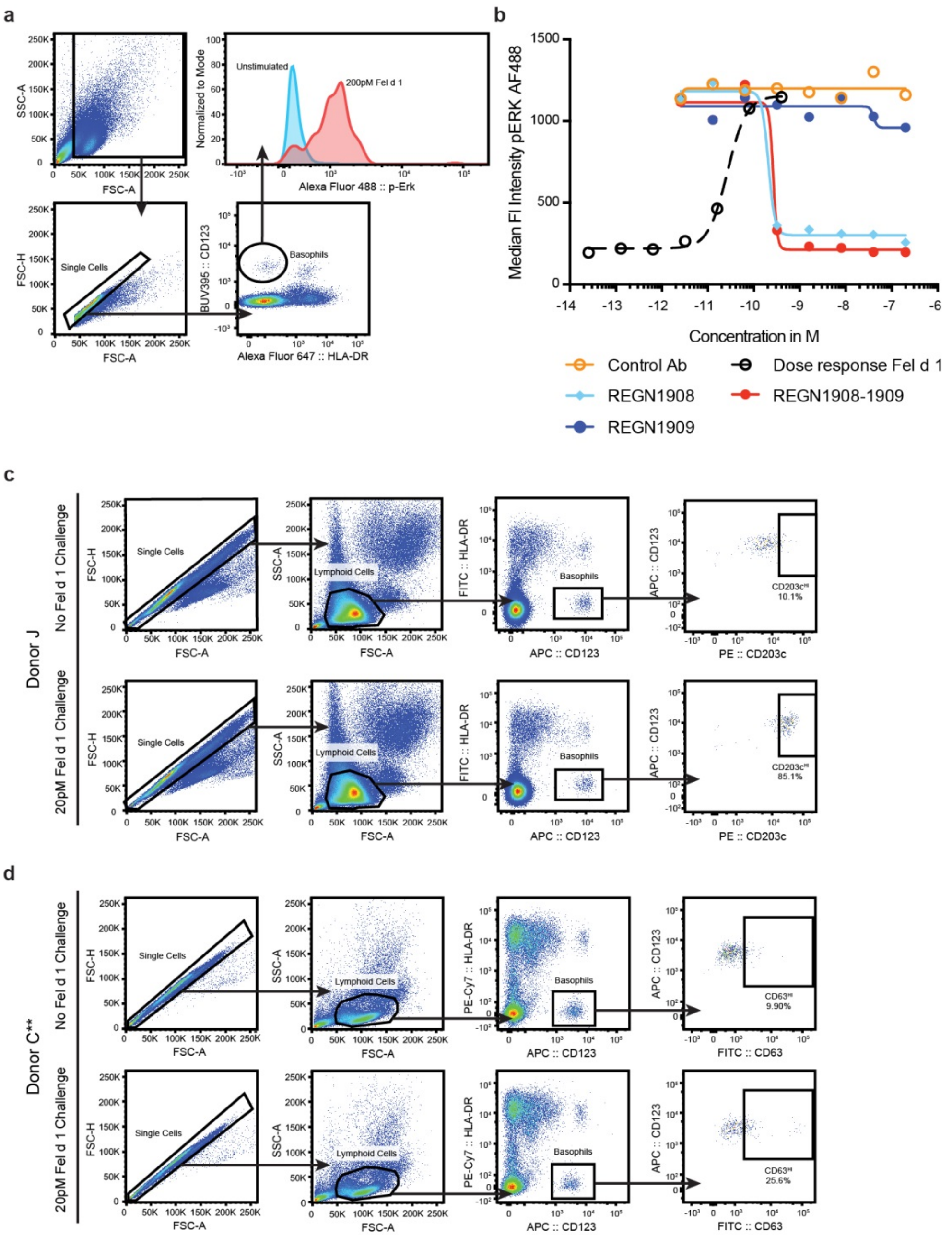

Supplementary Figure 5: Effector cell gating strategies. a Representative gating strategy used for the functional phosphoflow assay that measures phosphorylation of the kinase ERK in response to stimulation with natural Fel d 1 (nFel d 1). Basophils were defined as live (by forward scatter and side scatter), singlets, $CD123^+$, $HLA\text{-}DR^-$. Histogram shows phospho-ERK (pERK) staining within the basophil gate from one unstimulated sample (blue) and one sample stimulated with 200pM nFel d 1 (red). **b** Graph shows the median fluorescence intensity of the pERK response in 1 donor induced by a constant dose of 200pM of nFel d 1 in the presence of a dose response of the indicated antibodies. The black curve shows the pERK dose response to nFel d 1. Graph is representative of the data from all donors shown in Figure 2c. **c and d** Representative flow cytometry gating strategy for basophil activation test assays. Basophils were defined within the total PBMC population as singlets, lymphoid, $HLA\text{-}DR^-$, and $CD123^+$. Activation was characterized by increased surface expression of CD203c (**c**) or CD63 (**d**). To set a baseline level of basophil activation, conditions with no nFel d 1 challenge were used to draw $CD203c^{Hi}$ and $CD63^{Hi}$ gates containing 10% of the basophil population. Basophil activation in all other samples was measured by the percentage of basophils falling within these gates. Donor C, C*, C** represent independent donations from the same donor.

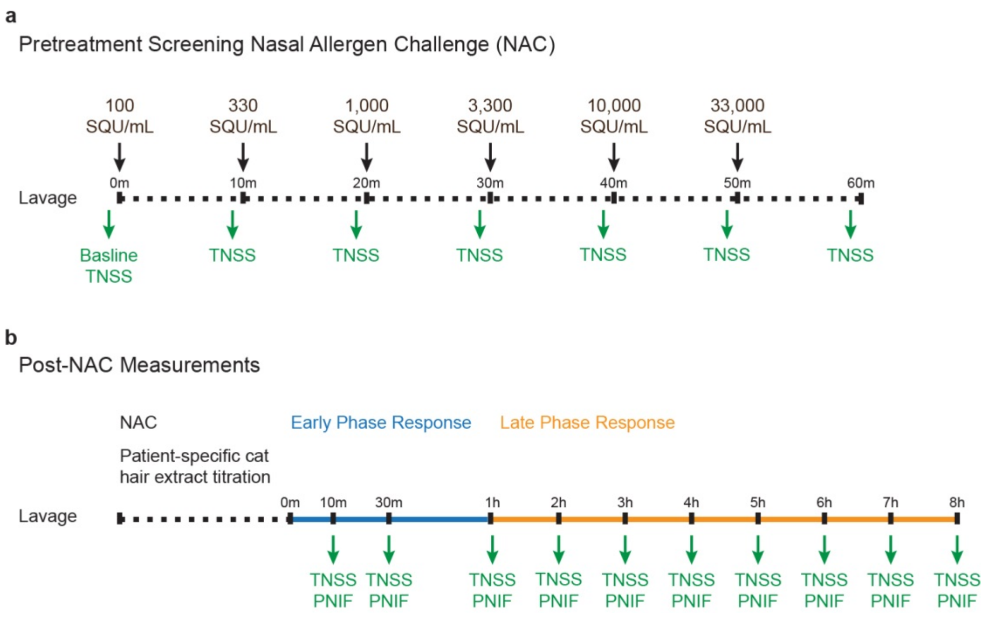

Supplementary Figure 6: Schematic of the Nasal Allergen Challenge procedure. a Increasing doses of cat hair extract were instilled intranasally every 10 minutes until Peak Total Nasal Symptom Score (TNSS) ≥7 was reached (on a 12-point scale). Upon TNSS ≥ 7, titration was stopped and the Early and Late Phase Responses were measured as depicted in (**b**). Eligible patients had Baseline TNSS ≤ 2, and reached Peak TNSS ≥ 7 within 60 minutes of Screening NAC initiation. **b** For each study visit, Day 8, 29, 57, and 85 after administration of REGN1908-1909 (600 mg, 1:1 ratio) or placebo, each patient was administered the patient-specific cat hair extract titration established during the Screening NAC, not exceeding the original maximal dose (regardless of whether TNSS ≥7 was reached at the study visit). To measure the Early Phase Response (designated as the first hour after allergen installation), patient TNSS, Peak Nasal Inspiratory Flow (PNIF) were measured at 10, 30 and 60 minutes, followed by every hour until hour 8.

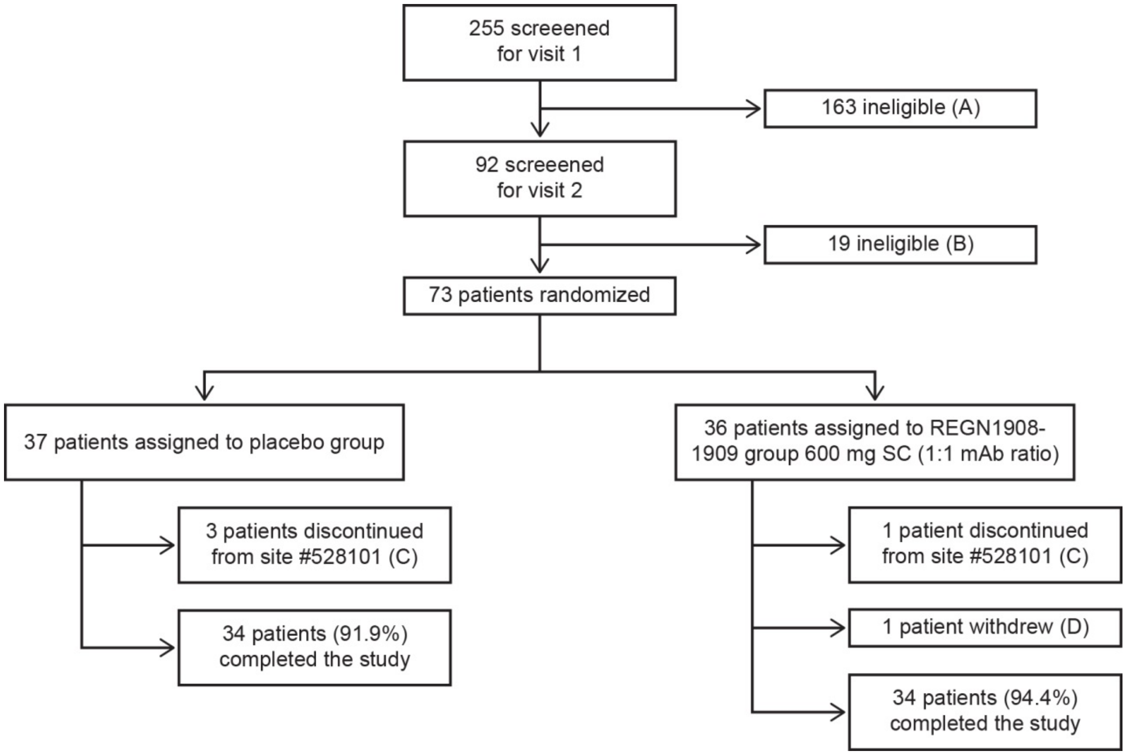

Supplementary Figure 7. Participant disposition. (A) The primary cause for screen 1 ineligibility was failure to confirm cat sensitization both by positive skin prick test (mean wheal diameter at least 3 mm greater than a negative control skin prick test) and/or allergen-specific IgE for cat dander and Fel d 1 (>0.35 kAU/l for both allergens) (B) The primary cause for screen 2 ineligibility was failure to achieve positive nasal allergen challenge (NAC) (defined as pre-challenge Total Nasal Symptom Score (TNSS) $\leq$ 2, and peak TNSS score $\geq$7 [on a 12-point scale] within 1 hour. (C) The decision to remove patients from site #528101 was made by the investigator/Sponsor due to noncompliance of the site. (D) One patient withdrew consent. Total patients treated: the safety analysis set was comprised of 37 patients (placebo) and 36 patients (REGN1908-1909). The full analysis set was comprised of 36 patients (placebo) and 34 patients (REGN1908-1909) because 3 treated participants did not have TNSS results on the day 8 visit. There were no discontinuations due to adverse event.

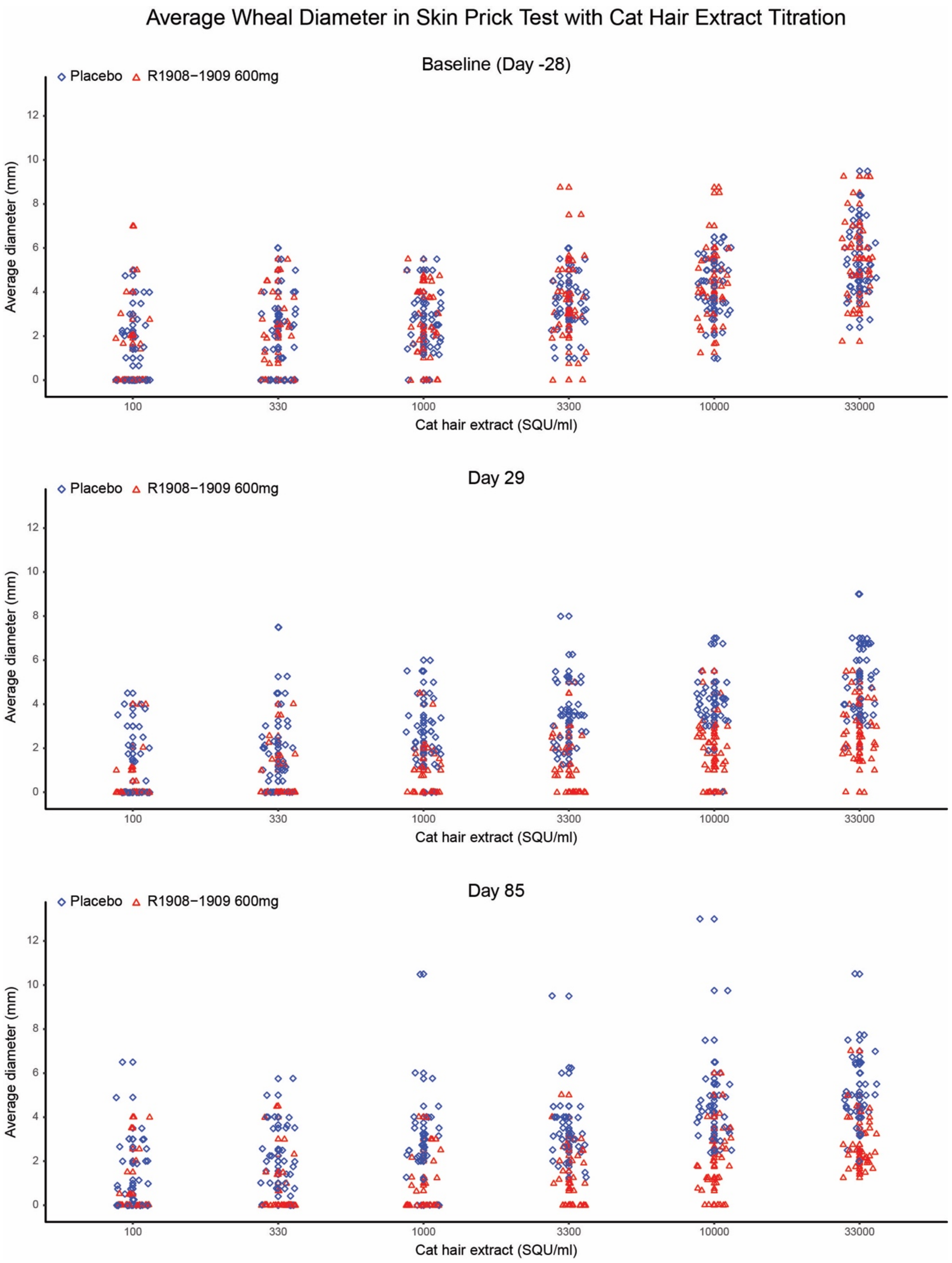

Supplementary Figure 8. Absolute Wheal Diameter for the Titrated Cat Hair Extract Skin Prick Test. For the titrated cat hair extract skin prick test (100-33,000 SQ-U/ml), average wheal diameter was measured 15 min post-application, as described in Methods. Individual titrated patient data are presented for Baseline, Day 29, and Day 85.
